# Formulation of low temperature mixed mode crack propagation behavior of crumb rubber modified HMA using artificial intelligence

**DOI:** 10.1038/s41598-025-08404-5

**Published:** 2025-07-01

**Authors:** Sepehr Ghafari, Mehrdad Ehsani, Sajad Ranjbar, Mohammad Nabi Nazari, Fereidoon Moghadas Nejad

**Affiliations:** 1 School of Built Environment, Engineering and Computing, Leeds Beckett University, Leeds, UK; 2 Department of Civil & Environmental Engineering, Amirkabir University of Technology (Tehran Polytechnic), Tehran, Iran

**Keywords:** HMA, Fracture, Mixed-mode loading, R-curve, Machine learning, Civil engineering, Mechanical properties

## Abstract

Determining mixed mode fracture parameters asphalt concrete mixtures remains an engineering challenge due to non-homogeneity and inelasticity of the material. In this research, a study was conducted to determine the low-temperature R-curves of unmodified and crumb rubber modified Hot Mix Asphalt (HMA) under mode I and mixed-mode (I/II) loading conditions. Single edge notched beam (SE(B)) testing was employed to collect data, and three key fracture parameters—cohesive energy, energy rate, and fracture energy—were extracted to represent different stages of fracture and crack propagation. Within the scope of this study, it was observed that for the AC 85/100 paving grade bitumen, a temperature of − 20 °C serves as a critical temperature, shifting fracture from quasi-brittle to brittle. At this temperature, the stable crack growth region in the R-curves significantly shrinks, causing abrupt specimen failure. The incorporation of 20% crumb rubber demonstrated favorable material characteristics, with a progressively rising R-curve even during the unsfi crack propagation phase. The central goal of this research is to establish prediction models for the mixed-mode (I/II) crack propagation parameters G_b_, G_f_, and G_i_. The features selected for modeling are G_b0_, G_f0_, and G_i0_ (mode I), percentage of crumb rubber, type of aggregate, binder content, nominal maximum aggregate size, temperature, and normalized offset ratio. Two dataset configurations were used: dataset 1 contains all entries, while dataset 2 excludes G_b0_, G_f0_, and G_i0_ (mode I). Five machine learning techniques, Regression, Multi-Gene Genetic Programming (MGGP), Support Vector Regression (SVR), Random Forest, and Artificial Neural Networks were employed to predict three key fracture parameters. Although slightly less accurate than SVR and Random Forest, MGGP offers the key advantage of yielding explicit mathematical expressions for crack propagation prediction. The R^2^ index for the MGGP model in Dataset 1 was 0.93 for G_b_, 0.94 for G_f_, and 0.92 for G_i_. For dataset 2, the indices were 0.89, 0.93, and 0.88, respectively.

## Introduction

Fracture of asphalt concrete mixtures in pavements rarely occurs in pure mode I during the service life^1^. Therefore, experimental investigations on mixed-mode (I/II) cracking of asphalt concrete and bituminous mixtures have gained wide attention in the past decade^2–4^. However, research on elastic–plastic-viscoelastic mixed-mode (I/II) fracture of asphalt concrete mixtures is still very scarce with controversial results^5^. Fracture toughness characterization of combined mode I and II loading conditions in bituminous mixtures has been carried out in several research^6,7^ so far. The toughness-based approach can be reliable in brittle fracture of bituminous mixtures where cracking is controlled by stresses, predominantly tensile and hydrostatic stress fields near the crack tip which is the case at extremely low temperatures. However, as the shearing component of loading is induced, the crack initiation and propagation phenomena will be governed by strains influencing the macroscale fracture toughness of the material^8^. In other words, in the case of small-scale yielding, the flow of energy into the crack tip can be described by the stress intensity factor which is independent of the size of the energy dissipation region, where the r^2^ singularity exists. However, the fracture of bituminous materials is not always k-controlled^9^ and large-scale yielding is observed^10^. Energy based analysis of mixed-mode (I/II) fracture of asphalt concrete mixtures has consequently received wide attention by researchers. Fakhri and Haghighat^11^ used the semi-circular bend SC(B) test configuration to investigate the mixed-mode (I/II) fracture resistance of these mixtures at intermediate to low temperatures. It was revealed in their study that the type of aggregate used, the performance grade (PG) of the binder, and air voids, have substantial effects on the fracture energy of the material within their test temperature scope. Pirmohammad and Abdi^12^ investigated the influence of support type and configuration on mode II fracture of asphalt concrete using both fracture toughness and fracture energy. They obtained the fracture energy magnitudes for various support types with different frictions from the load–displacement curves. It was concluded in their research that regardless of the support type, increasing the temperature reduced the mode II fracture energy of the mixtures. Zarei and Kordani^13^ investigated the effect of angular cracks on mode I and mode II fracture of asphalt concrete mixtures at intermediate temperatures using experimental and extended finite element methods. The SC(B) test protocol was used in their work and it was assumed that the viscoelastic matrix of the mixture dissipates the work done by the load into thermal energy. Hence, they used the fracture energy concept to study the fracture behavior and effect of the angular cracks. It was found that the presence of coarse aggregate in the ligament increased fracture energy and reduced the brittle fracture tendency of the mixtures. In recently published research, Ghafari and Moghadas Nejad^14^ determined low-temperature fracture resistance curves (R-curves) of asphalt concrete mixtures in mixed-mode (I/II) conditions. In this fashion, the total fracture regime of the mixtures could be mapped and investigated. Effect of binder content, aggregate type, temperature, and mode mixity were demonstrated on crack blunting, initiation, and propagation phases of fracture.

It can be inferred from the literature that mixed-mode (I/II) fracture investigations on asphalt concrete mixtures are still not as broad as that for pure mode I impacting mixture design for sustainable pavements. Codes such as AASHTO TP105^15^ or MnRoad 2014^16^ have presented instructions on mode I fracture energy determination of asphalt concrete mixtures while the effect of mode II loading is not considered.

One of the primary reasons the mixed-mode (I/II) behavior of asphalt mixtures has not been thoroughly investigated is due to the inherent challenges and the need for high precision in conducting mixed-mode (I/II) tests. On the other hand, the rapid growth of computational power has led to the widespread adoption of computational intelligence (CI) machine learning (ML) methods across various engineering applications^17^. This increase in computational capabilities has driven the development of more efficient algorithms and techniques, such as Artificial Neural Networks (ANN) and genetic programming, enabling enhanced performance and accuracy, and making these methods integral to solving complex engineering challenges^18,19^. Accordingly, a promising solution lies in employing CI-based methods and regression analysis to develop predictive models for the mixed-mode (I/II) behavior of asphalt mixtures allowing researchers to gain valuable insights into mixed-mode (I/II) properties and performance without the complexities of extensive physical testing, thereby saving time, resources, and effort. In pavement engineering, researchers have utilized machine learning methods to create predictive models. For example, Talebi et al.^20^ developed machine learning (ML) models to predict the fracture load of asphalt mixtures with high accuracy. They used RReliefF for feature selection and trained support vector machine, extra tree, and gradient boosting regressors on 675 experimental data points. Ensemble techniques further improved prediction accuracy, achieving over 91% accuracy. Their study highlights the effectiveness of ML in modeling complex material behaviors, offering enhanced precision over traditional methods for infrastructure assessment. Majidifard et al.^21^ conducted a study using Gene Expression Programming (GEP) to predict the fracture energy in asphalt mixtures, achieving an R^2^ value of 0.96. They collected a dataset of experimental data on the fracture energy of asphalt mixtures, including information on the useful temperature interval, low-temperature performance grade, percentage of asphalt content, nominal maximum aggregate size, percentage of reclaimed asphalt pavement, percentage of reclaimed asphalt shingles, gradation type, aggregate type, crumb rubber content, and testing temperature. This demonstrated that GEP can effectively predict asphalt mixture fracture characteristics. In their 2022 study, Mirzaiyanrajeh et al.^22^ developed a prediction model for low-temperature fracture energy of asphalt mixtures using a machine learning approach, analyzing 14 input parameters such as high/low-temperature PG binder grade, reclaimed asphalt for combined gradation, voids in mineral aggregates, asphalt film thickness, mixture maximum theoretical specific gravity, mixture bulk specific gravity, and aggregate bulk specific gravity. The model’s performance was evaluated using the coefficient of determination (R^2^), with results showing SVEM = 0.90, GEP = 0.68, and ANN = 0.80, indicating varying levels of accuracy among the different models in predicting fracture energy. In 2023, Nguyen et al.^23^ explored machine learning techniques to predict the Cracking Tolerance Index (CTIndex) of asphalt concrete containing reclaimed asphalt pavement. The study considered eight key input parameters: aggregate content passing the 4.75 mm sieve, aggregate content passing the 0.075 mm sieve, bitumen content, penetration at 25 °C, flash point, softening point, reclaimed asphalt pavement content, and rejuvenator content. The model’s predictive performance was measured, with R^2^ values of 0.91 for KNN, 0.96 for MARS, and 0.97 for RF, indicating a strong capability to accurately estimate the CTIndex. In addition, Nasr et al.^24^ evaluated the substitution potential of Styrene–Butadiene–Styrene (SBS) with crumb rubber-polypropylene blends as asphalt binder and mixture modifiers. The study analyzed four input parameters: crumb rubber, polypropylene, Styrene–Butadiene–Styrene (SBS), and moisture condition. The output focused on the fracture energy ratio, with the Radial Basis Function Neural Network (RBFNN) model demonstrating a high prediction accuracy, reflected in an R^2^ value of 0.95. In a separate research, Ghafari et al.^25^ attempted to predict the R-curve of asphalt mixtures in mode 1, utilizing both ANN and MGGP. The findings revealed that ANN outperformed the MGGP method, as the latter lacked the necessary accuracy to predict the R-curve reliably. However, it is worth noting that using ANN has its drawbacks, as it operates like a black box and does not provide a clear relationship that engineers can easily interpret and utilize. The model takes into account the, low-temperature performance grade of the binder, binder content, crumb rubber content, test temperature, maximum aggregate size, and time as input variables and predicts cumulative fracture energy and crack length extension as output variables. The R-square equaled 0.87 in this method.

In the reviewed studies, the primary focus has often been on predicting the failure energy of asphalt mixtures in mode I using machine learning techniques. However, several important aspects have been underemphasized. Firstly, fracture energy alone is insufficient to fully characterize the fracture behavior of asphalt. It fails to provide comprehensive information about the process of crack propagation, which is crucial for understanding the material’s long-term performance and durability. Additionally, there has been a disproportionate focus on mode I fracture, with less attention given to mixed-mode (I/II) fracture behavior, which is more representative of real-world conditions where multiple failure modes can occur simultaneously. Moreover, the widespread use of black-box machine learning models in these studies, while effective in prediction, often overlooks the underlying mathematical relationships between asphalt mixture parameters and their failure behavior.

In this study, the fracture behavior of asphalt mixtures will be investigated using three key parameters: cohesive energy (G_b_), energy rate (G_i_), and fracture energy (G_f_). The analysis will be conducted under both mode I and mixed-mode (I/II) loading conditions to provide a comprehensive understanding of the material’s performance. Linear regression and multi-gene genetic programming methods will be employed to develop prediction models. These methods will be used to model and predict the fracture behavior based on the parameters. For this purpose, comprehensive experimental investigations involving SE(B) testing were conducted on unmodified and crumb rubber modified asphalt concrete to establish low-temperature R-curves for these mixtures under mode I and mixed-mode (I/II) loading conditions.Three key fracture parameters )G_b_, G_i_, and G_f_) were extracted from the R-curves to represent distinct phases of fracture and crack propagation. The tests were performed at various temperatures ranging from + 5 °C to − 20 °C. Modified mixtures incorporated 10% and 20% crumb rubber powder, while reference mixtures contained no crumb rubber. Two types of lime and siliceous aggregates with different gradations were used in the mixtures. Pure mode I loading and mixed-mode (I/II) loading conditions with varying shearing component contributions were applied, enabling the investigation of the transition of R-curve quantitative parameters from mode I to mixed-mode (I/II).

## Research objective

This study aims to formulate and develop predictive models for the mixed-mode (I/II) output variables G_b_, G_f_, and G_i_. The dataset used in this research consists of two main subsets:

- Dataset 1 includes G_b0_, G_f0_, and Gi_0_ (mode I), percentage of crumb rubber, aggregate type, percentage of bitumen, maximum aggregate size, temperature, and normalized offset ratio.
- Dataset 2 predicts G_b_, G_f_, and G_i_ without considering G_b0_, G_f0_, and G_i0_ (mode I).

**Fig. 1:**
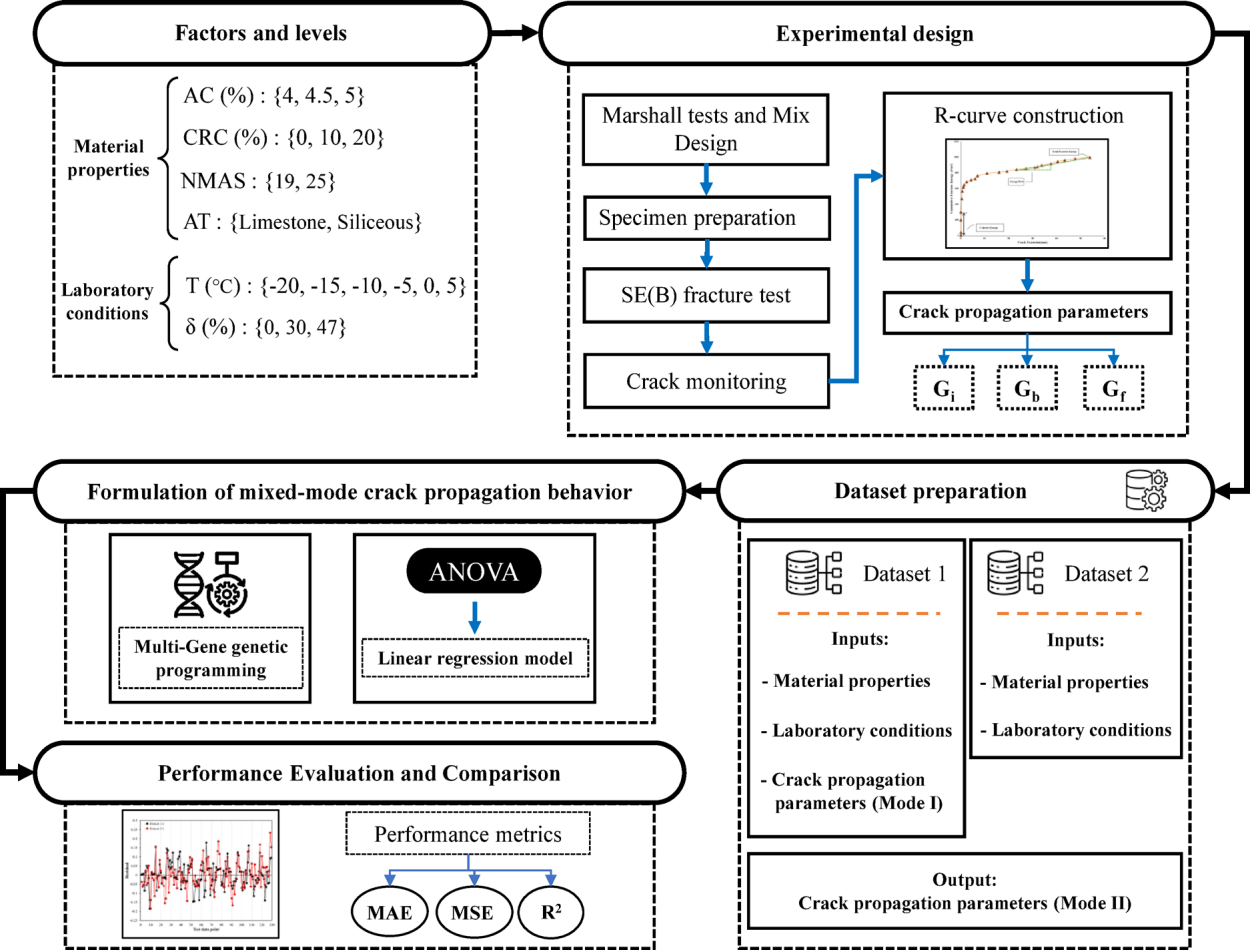
Schematic of the methodology employed.

Using linear regression and MGGP, distinct predictive models were developed for G_b_, G_f_, and G_i_ in the mixed-mode (I/II). These models outperform conventional machine learning approaches by establishing clear relationships between input and output parameters, making them easily applicable to researchers. The developed models underwent thorough validation and evaluation, and the most effective ones are presented for use. Figure 1 provides a schematic of the methodology used in this study.

## Materials and methods

### Materials

**Fig. 2:**
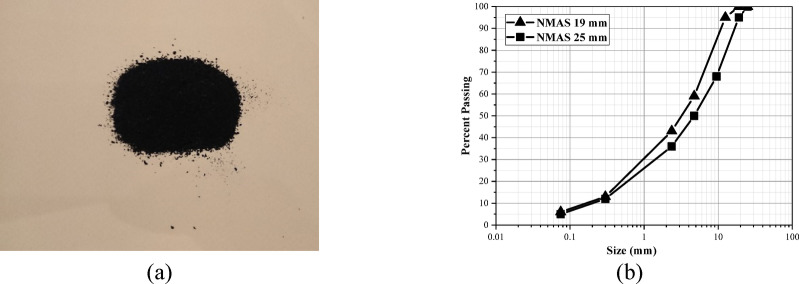
(**a**) crumb rubber powder, and (**b**) gradations curves of the mixtures.

Two types of asphalt concrete mixtures were prepared in this study: crumb rubber modified and unmodified. Limestone aggregate was used for both types of mixtures with two nominal maximum aggregate sizes (NMAS): 19 mm and 25 mm (as shown in Fig. 2). These gradations represent typical local designs for binder and wearing courses. Aggregate physical properties are summarized in Table 1. The base binder used in the mixtures was AC 85/100, and its specifications are provided in Table 2. To determine the optimum binder content for each mixture type, Marshall testing was conducted. The results indicated an optimum binder content of 4.5% for mixtures with an NMAS of 19 mm and 4.2% for mixtures with an NMAS of 25 mm. Based on these findings, three binder content levels—4%, 4.5%, and 5%—were considered for all mixtures to assess the influence of binder content on their performance under different loading modes.

**Table 1:** Physical properties of the lime and siliceous aggregate.

Property	Standard	Limestone aggregate	Siliceous aggregate
LA abrasion loss	AASHTO T96-02	20	19
Fractured in one face	ASTM D5821-13	> 98	> 87
Fracture in two faces	ASTM D5821-13	> 98	93
Flakiness	BS 812-103.1	5	20
Coating of aggregate	AASHTO T184	98	95
Sodium solphate loss	AASHTO T104-99	1.8 (fines) 0.7 (coarse)	2.8 (fines) 0.4 (coarse)

**Table 2:** Rheological properties of the binder.

Property	AC 85/100	Test method
Penetration at 25 °C, 0.1 mm	89	ASTM D5
Softening point (°C)	50.8	ASTM D36
Ductility (15 °C, cm)	> 100	ASTM D113
Flashing point (°C)	290	ASTM D92
Density	1.02	ASTM D70
Kinematic viscosity (Centistoke, 120 °C)	790	ASTM D2170
Kinematic viscosity (Centistoke, 135 °C)	370	ASTM D2170
Kinematic viscosity (Centistoke, 160 °C)	130	ASTM D2170
DSR (G*/sin(δ), kPa)	1.29	ASTM D7175

Crumb rubber modified asphalt concrete mixtures were produced using 10% and 20% crumb rubber by weight of the base binder. The crumb rubber was obtained in the form of fine powder (Fig. 2) with a maximum particle size of 0.6 mm (#30) through the grinding of recycled waste tires. Before incorporating crumb rubber into the base binder, a warm-mix additive (Sasobit) was added to the neat bitumen at a dosage of 3% by weight of the binder. The mixture was then subjected to a thorough mixing process at a temperature of 160 °C for 15 min using a rotational velocity of 6000 rpm. Next, the binder temperature was gradually raised to 180 °C, and the crumb rubber powder was introduced into the mixture. The crumb rubber powder was incorporated into the binder using a mixing speed of 2000 rpm for 30 min, resulting in a uniform and well-blended rubberized binder. In the final step, the rubberized binder was combined with preheated aggregate and compacted at 170 °C to produce asphalt concrete slabs.

**Fig. 3:**
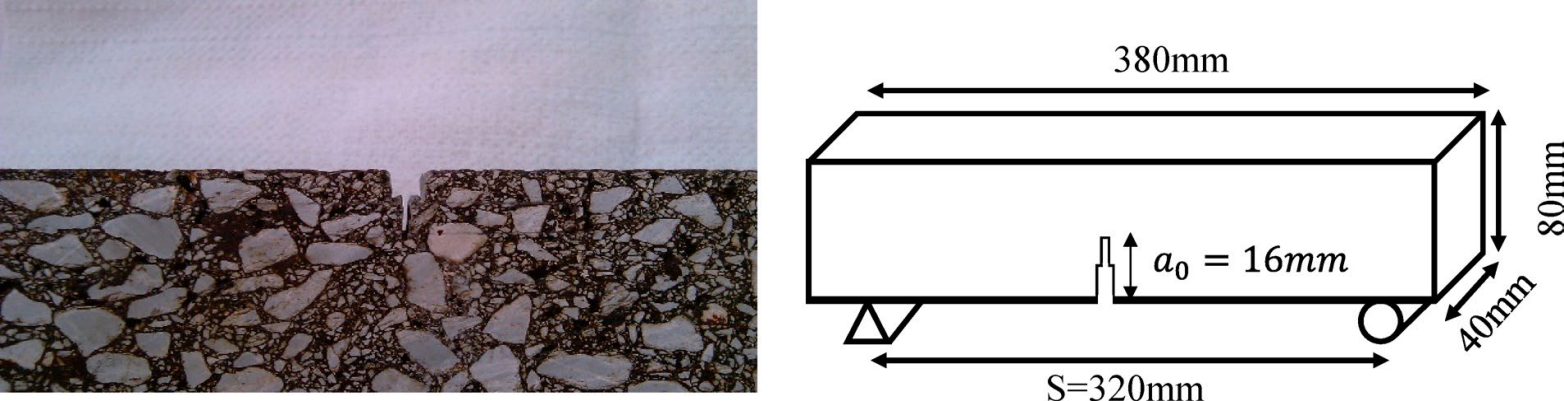
Details of the fabricated notch in AC beam specimens.

Asphalt concrete slabs were compacted using slab compactor equipment to an air void ratio of 4% ± 1%. The beam specimens were cut from the slabs after cooling the slabs for at least 7 h from compaction. Beam dimensions for mode I and mixed-mode (I/II) experiments were selected based on the instructions in ASTM E1820-20b^26^. Hence, each beam was cut to a total length of 380 mm resulting in a span length (S) of 320 mm for the fracture tests. The height (width) of the beams equaled 80 mm (w) with a thickness of 40 mm. A mechanical notch was fabricated in each beam with a constant notch to width ratio of 0.2 resulting in a notch length of 16 mm. The notch was fabricated in a two-stage procedure. First, a length of 8 mm (half of the total notch length) was cut by a water cooled saw with a blade thickness of 5 mm. The remaining 8 mm was then cut by a handsaw having a blade thickness of less than 1 mm for the maximal mitigation of blunting occurrence possibility at the notch tip (Fig. 3)^27^.

A water based white paint was applied to the two surfaces of the beams to induce desirable contrast between the growing crack and the uncracked surfaces during the tests.

### SE(B) tests in mode I and mixed-mode (I/II)

The SE(B) test configuration offers the flexibility to achieve various loading conditions. Due to its simple stress fields and insignificant end effects, it has been widely employed by researchers^28^ to investigate fracture characteristics of asphalt concrete.

**Fig. 4:**
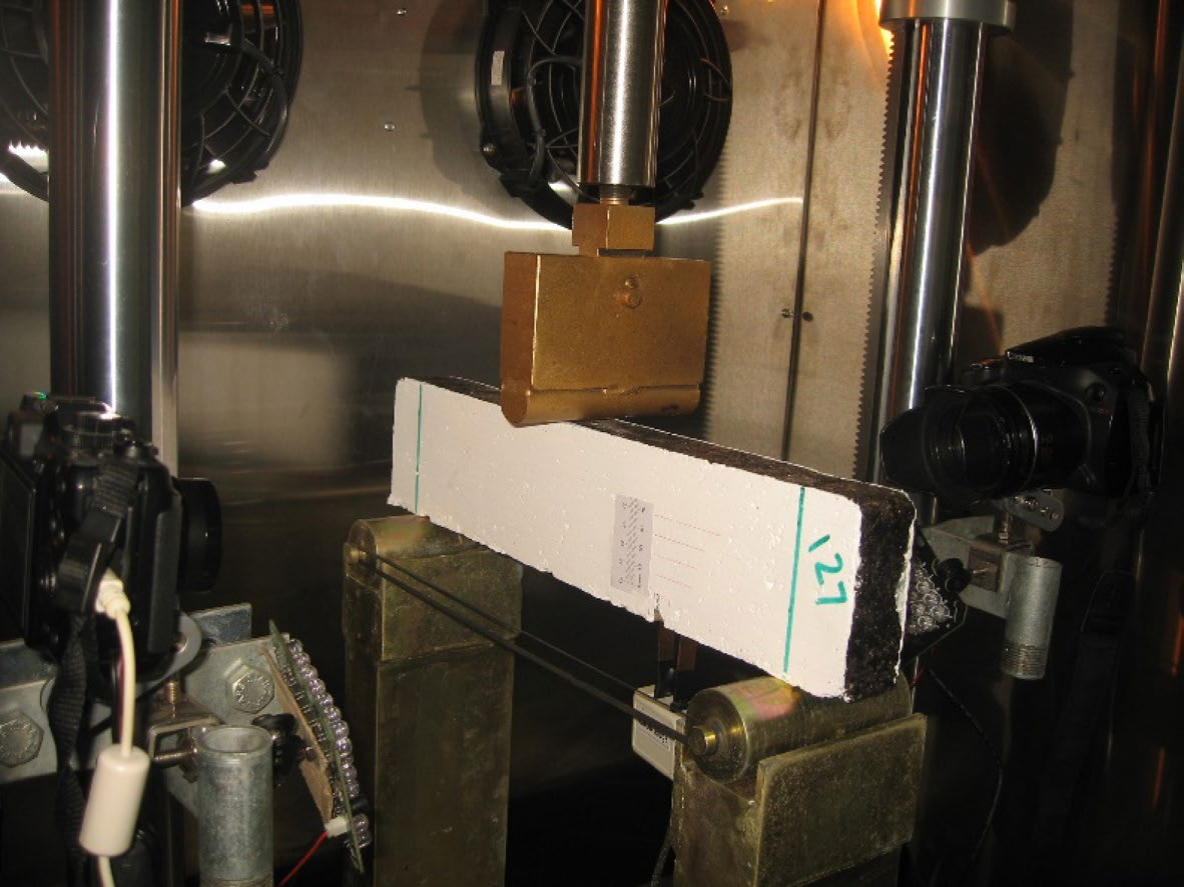
Mixed-mode (I/II) SE(B) test setup.

All tests were conducted within the environmental chamber of a universal testing machine (UTM). The asphalt beam specimens were placed in the chamber at least four hours before each test to ensure uniform temperature distribution throughout the specimens. Both mode I and mixed-mode (I/II) tests were performed using the setup illustrated in Fig. 4. A fixture was designed based on ASTM E1820-20b guidelines, taking into account the available space in the loading frame and the beam dimensions. The fixture provided a span length of 320 mm and a width of 100 mm. Two roller supports were designed with a diameter of 40 mm. Vertical load was applied to the top surface of the beams at the centerline of the beam span using a half-roller with a radius of 10 mm (w/8). The UTM possessed a loading capacity of 25 kN, and the loading location remained constant for all experiments. Load-line displacements (LLD) and the axial force were recorded using the linear variable differential transformer (LVDT) of the actuator. Additionally, a CMOD gauge was developed based on ASTM 1820-20b and was mounted on the bottom surface of the beams at the notch centerline to measure crack opening displacements during beam loading.

**Fig. 5:**
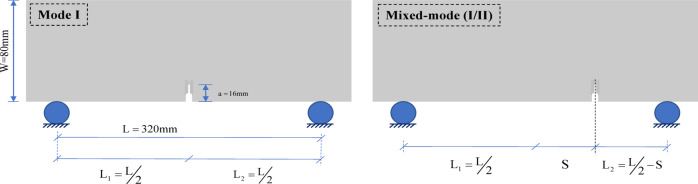
Schematics of mixed-mode (I/II) generation by introducing notch offsets.

Mixed-mode (I/II) loading conditions were generated by modifying the notch location using offsets from the centerline of the beam span. Three notch offsets were considered for the mixed-mode (I/II) states which generate different contributions of the bend and shear components. The normalized notch offset (γ) parameter can be characterized by Eq. (1):

1 $$\gamma = \frac{2 \times S}{{l_{1} + l_{2} }}$$

where $S$= Lateral notch offset distance from the centerline of the beam (mm); ${l}_{1}, {l}_{2}$=Distance between the centerline of the beam and the supports (mm); Schematics of mode mixity generation by introducing notch offsets can be seen in Fig. 5 and the corresponding values within the scope of this research are presented in Table 3.

**Table 3:** Notch locations for generating mode I and mixed-mode (I/II) loading conditions.

Notch offset, S (mm)	Normalized offset ratio (γ)	l_1_ (mm)	l_2_ (mm)
0	0	190	190
48	0.3	190	142
75.2	0.47	190	114.8

Crack growth was monitored during each test using two high-resolution digital cameras (18 and 20 megapixels) positioned on either side of the beam. The cameras captured images of the notch at a rate of 5 and 15 frames per second, respectively. The images were analyzed using the open-source imageJ software to measure the crack growth increment. A custom script was developed to synchronize loading time with the EXIF data embedded in the captured images. The average crack extensions from both beam surfaces were used to construct the R-curves.

Before commencing the test, a preload of 0.1 kN was applied to ensure that the beam was fully seated on the supports. The tests were conducted under displacement control at a loading rate of 5 mm/min, as recommended by Braham and Buttlar^29^.

### Crack propagation parameters and the R-curve method

Fracture resistance curves offer a powerful framework for understanding and quantifying how asphalt mixtures resist cracking as damage accumulates. Unlike single-parameter fracture analysis that relies on failure- associated parameters, R-curves describe the evolution of crack resistance throughout the entire fracture process^30,31^. This makes them especially valuable for characterizing modified asphalt mixtures—such as polymer or rubber-modified—that may exhibit complex fracture behavior. By plotting energy-based parameters such as the J-integral or fracture energy against crack extension, R-curves provide insight into both the material’s resistance to crack initiation and its capacity to resist further propagation. This distinction is critical, as materials with similar initial toughness can behave very differently once cracking begins. R-curves derived from common test methods such as the single-edge notched beam (SE(B))^32^, semi-circular bending (SCB), and disk-shaped compact tension (DCT) tests have been used for fracture characterization of asphalt mixtures^33–35^.

Three quantitative parameters characterizing the crack propagation behavior of asphalt concrete mixtures are identified and extracted in this study. These parameters include the energy required to blunt the crack tip (G_b_), which is also known as the cohesive energy, fracture energy (G_f_), and the slope of the R-curve in the post-peak phase (G_i_), which is also referred to as the energy rate, indicating the rate of energy dissipation in the unstable crack propagation zone. To determine these parameters, fracture resistance curves of the mixtures in mode I and mixed-mode (I/II) need to be generated.

In this research, R-curves are constructed by plotting the cumulative fracture energy (G) as a function of crack extension (Δa). The cumulative fracture energy represents the energy dissipated in deflecting the asphalt concrete beam as the load is applied. The crack extensions are obtained by analyzing the images captured during each load increment. It should be noted that the cumulative fracture energy itself encompasses multiple components, such as elastic strain energy, dissipated cohesive energy for forming crack surfaces, and creep energy. For all experiments, LLD-based cumulative fracture energy was used, and its calculation was based on RILEM TC-50 FMC^36^, with the self-weight of the beams being disregarded.

**Fig. 6:**
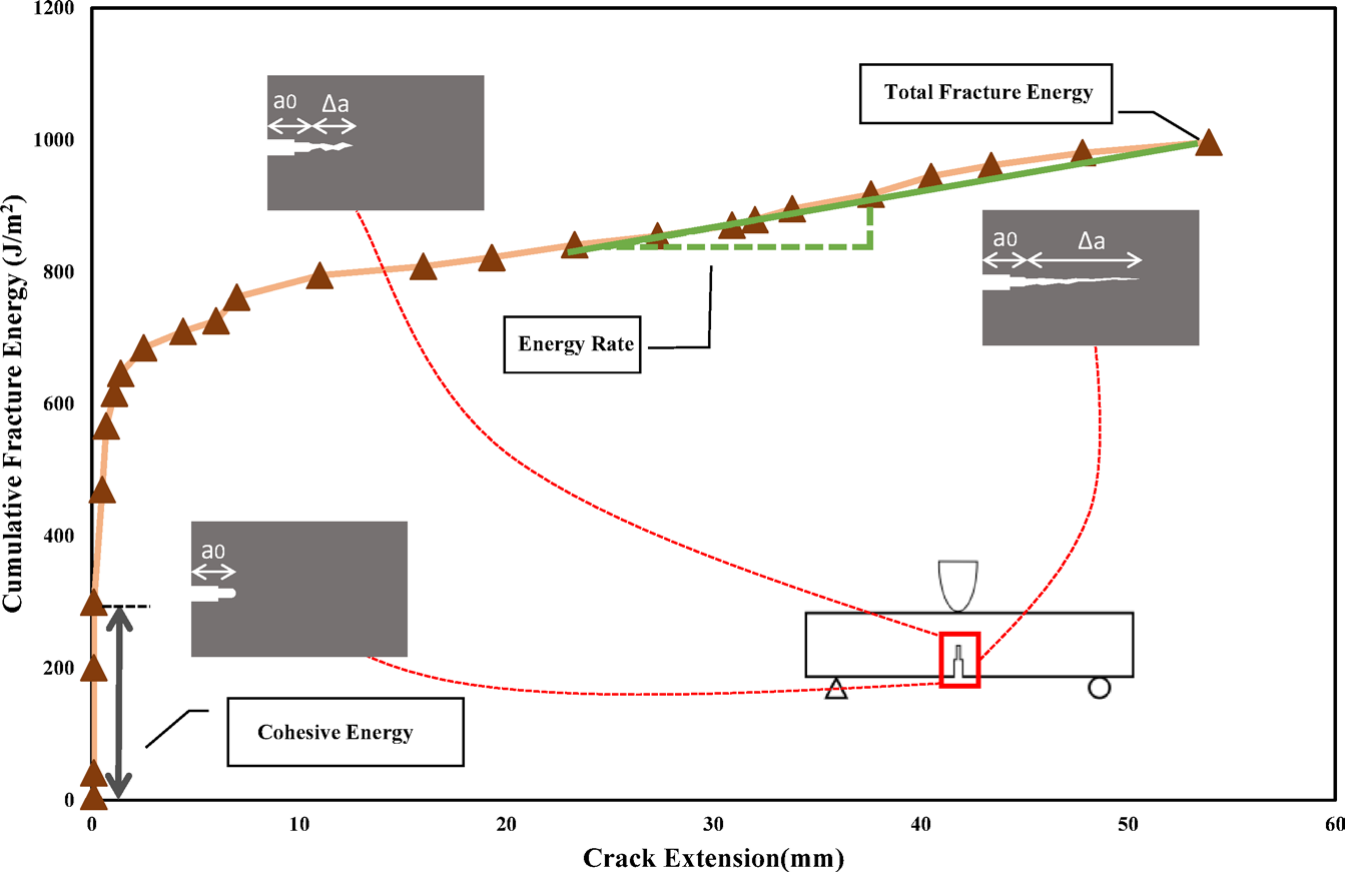
Sample R-curve for crumb rubber modified mixtures tested at − 20 °C and the quantitative fracture parameters.

A sample mixed-mode (I/II) R-curve for the mixture with 20% crumb rubber incorporation is presented in Fig. 5, representing a generic example. R-curves commence with a vertical segment corresponding to blunting the crack tip. This phase, also known as the cohesive portion of the R-curve, indicates the energy dissipated in forming cohesive surfaces to initiate a visible crack. The point where the vertical portion of the R-curve diverges from the y-axis corresponds to the height of the vertical segment, denoted by G_b_, as shown in Fig. 6.

A visible growing crack is initiated at the separation point. The mixture can undergo stable or unstable crack growth in this stage leading to a dominating unstable propagation zone. The unstable crack propagation zone proceeds with the stable crack growth and continues until failure of the asphalt concrete beam. The right-most point on the R-curves therefore indicate the total fracture energy (G_f_) and the overall slope of the R-curve (G_i_) is indicative of the energy dissipated per unit extension of the crack in the post-peak phase. The G_i_ can also be formulated as in Eq. (2)^37^:

2 $$G_{i} = \frac{{G_{f} - G_{b} }}{{\Delta a_{t} }}$$

where ${G}_{i}$= The slope of the R-curve in the unstable propagation zone or energy rate (J/m^3^), ${G}_{f}$= Total fracture energy (J/m^2^), ${G}_{b}$= The energy required to blunt the crack tip (J/m^2^), $\Delta {a}_{t}$= Total crack extension (mm).

### Machine learning framework

#### Dataset

The main objective of this study is to develop prediction models for the mixed-mode (I/II) crack propagation parameters (output variables) G_b_, G_f_, and G_i_. The input facors considered in the analysis include crumb rubber content (CRC), type of aggregate (AT), binder content (B), nominal maximum aggregate size (NMAS), temperature (T), and normalized notch offset (γ). In accordance with a full factorial experimental design, a total of 648 experiments were conducted to evaluate the interactions among mentioned input factors. The levels and specific values of these factors are detailed in Table 4.

**Table 4:** Factors and their levels.

Factor	Abbreviation	Unit	Range of variation					
			Level 1	Level 2	Level 3	Level 4	Level 5	Level 6
Crumb rubber content	CRC	%	0	10	20	–	–	–
Binder content	BC	%	4	4.5	5	–	–	–
Temperature	T	°C	− 20	− 15	− 10	− 5	0	5
Normalized notch offset	γ	%	0	30	47	–	–	–
Aggregate type	AT	–	Limestone	Siliceous	–	–	–	–
Nominal maximum aggregate size	NMAS	mm	19	25	–	–	–	–

In this study, mode I includes three input variables: G_b0_, G_f0_, and G_i0_. Determining these variables requires conducting experiments, which can be costly. To address this, two different datasets were generated, one of which include G_b0_, G_f0_, and G_i0_ incorporating mode I fracture features, while the other excludes them. These datasets are outlined in Table 5.

**Table 5:** Datasets description.

Variable ID	Dataset 1–1	Dataset 1–2	Dataset 1–3	Dataset 2–1	Dataset 2–2	Dataset 2–3
Input variables						
G_f0_ (Pa)	✓					
G_b0_ (Pa)		✓				
G_i0_ (Pa)			✓			
γ	✓	✓	✓	✓	✓	✓
B (%)	✓	✓	✓	✓	✓	✓
CRC (%)	✓	✓	✓	✓	✓	✓
T (°C)	✓	✓	✓	✓	✓	✓
AT	✓	✓	✓	✓	✓	✓
NMAS (Hz)	✓	✓	✓	✓	✓	✓
Output variables						
G_f_ (Pa)	✓			✓		
G_b_ (Pa)		✓			✓	
G_i_ (Pa)			✓			✓

Table 6 presents descriptive statistics and the significance of both input and output variables, including minimum, maximum, average, and standard deviation values.

**Table 6:** Descriptive statistics of variables.

Variable ID	Average	Standard deviation	Minimum	Maximum
Main input variables				
G_f0_ (Pa)	672.5	230.9	203.7	1343.5
G_b0_ (Pa)	191.9	89.7	36.9	456.7
G_i0_ (Pa)	1.6	0.8	0.2	4.5
γ (%)	0.26	0.2	0	0.47
B (%)	4.5	0.4	4	5
CRC (%)	10	8.2	0	20
T (°C)	− 7.5	8.5	− 20	5
AT	–	–	0	1
NMAS (mm)	–	–	19	25
Output variables				
G_f_ (Pa)	1085.6	559.5	203.7	3060.8
G_b_ (Pa)	294.8	203.4	36.9	1487.8
G_i_ (Pa)	2.4	1.7	0.2	11.2

Transformation is an effective statistical method for converting non-normal data into a normal distribution. However, some issues may arise, such as the non-normality of errors and heteroscedasticity, which violate the main assumptions of Ordinary Least Squares (OLS). The normality of the response data significantly impacts these issues and the performance of the regression model. Therefore, the Box-Cox transformation can be applied to create a more efficient and credible prediction model. A common form of this transformation is as follows:

3 $$Y_{i}^{ * } = \left\{ {\begin{array}{*{20}c} {(Y_{i} - \omega )^{\lambda } } & {{\text{if}}} & {\lambda \ne 0} \\ {\ln (Y_{i} - \omega )} & {{\text{if}}} & {\lambda = 0} \\ \end{array} } \right.$$

where $Y_{i}^{ * }$ is the transformed value of $Y_{i}$.$\omega$ is a shift factor that is added when Y is zero or negative. $\lambda$ is the value of power. The optimum value of $\lambda$ is unknown and is determined in an optimization process based on the results of normality tests on transformed data.

Another crucial step in data preprocessing involves scaling. Learning methods often rely on objective functions that utilize Euclidean distance. If the range of certain data is substantially larger than that of others, the algorithm may overly prioritize those data points, which is undesirable. To address this issue, it is necessary to scale all input data to a standardized interval. Equation (4) demonstrates the method for scaling the input variables^38,39^.

4 $$S_{i} = \frac{{R - R_{\min } }}{{R_{\max } - R_{\min } }}$$

where ${S}_{i}$ represents the scaled value of the ith variable ${R}$ corresponds to the measured value, and ${R}_{max}$, and ${R}_{min}$ correspond to their minimum and maximum, respectively. By utilizing this relationship, we can effectively scale the input variables of both datasets within the range of 0–1.

To train and validate the model, the data was randomly split into two distinct parts: 80% for the training set and 20% for the testing set. The trained model and its unknown parameters are determined using training data. The model is then evaluated using test data.

#### Linear regression model

Regression analysis is a statistical method used to establish a relationship between input and output data. Linear regression is the most common form of regression analysis, generating a linear equation as follows:

5 $$y = w_{0} + w_{i} x_{i} + \varepsilon$$

where y is the model output, x_i_ represents the input variables, $\varepsilon$ is the prediction error, and $w$ is the model coefficients with $w_{0}$ being the intercept of the model. The coefficients $w_{i}$ indicate the correlation between each of the input variables and the output.

The selection of variables with significant effect on outputs is very important for generating an efficient prediction model. Analysis of variance (ANOVA) is used here to determine the effect of input variables on outputs by calculating the p-values. Generally, factors with p-values greater than 0.1 are regarded as insignificant, while those with p-values between 0.1 and 0.05 exhibit minor significance. Factors with p-values below 0.05 are considered to have a significant influence on the response, reflecting a high level of statistical significance^40–42^. In this study, the models were developed using the highly significant factors (*p* value < 0.05).

factors which have *p* values less than 0.05 are considered as significant factors. while insignificant variables are those with a *p* value of greater than 0.1.

#### Multi-Gene genetic programming

**Fig. 7:**
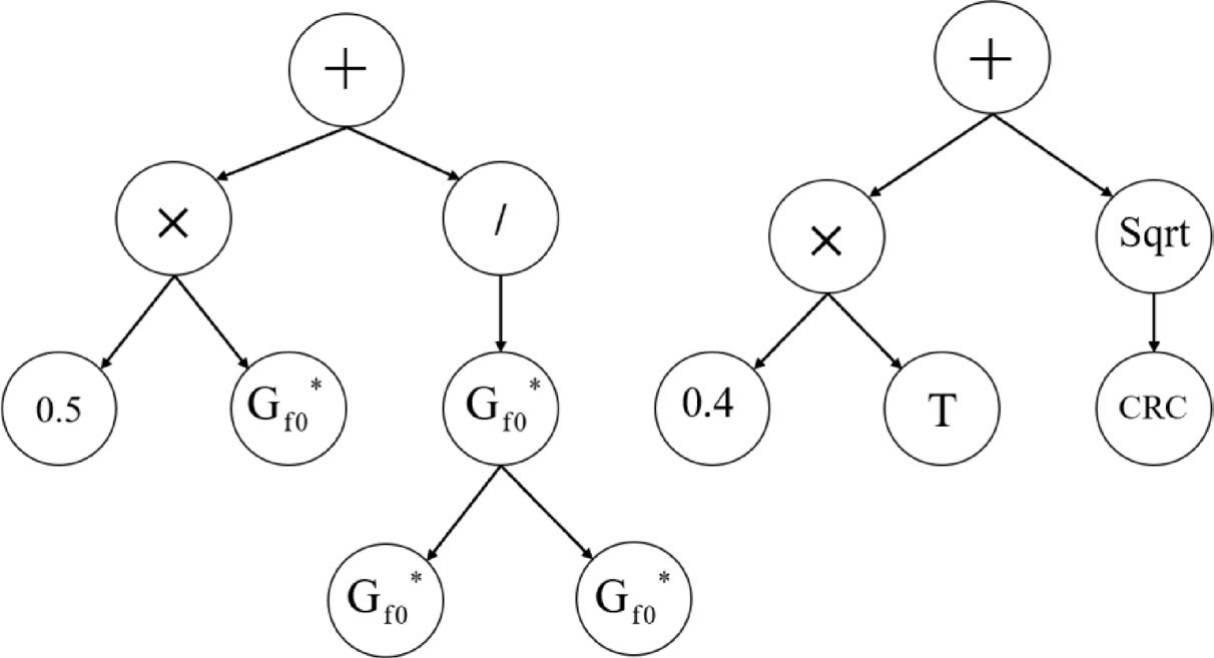
A sample model developed by MGGP.

Conventional machine learning methods often function as black boxes, predicting the target variable solely from the input variables. The complexity of the relationship between input and output in these conventional methods can make their utilization seemingly impossible. However, every machine learning method essentially boils down to an optimization problem^43^, aiming to minimize the error between the model and the data. By leveraging metaheuristic algorithms and formulating an optimization problem^44^, it becomes feasible to establish a straightforward mathematical relationship between the input and output variables, leading to enhanced interpretability and simplicity in the model’s representation^45,46^. Multigene genetic programming is a powerful method that establishes a straightforward and highly precise mathematical relationship between input and output variables using the genetic optimization algorithm. Figure 7 presents a sample model developed using the MGGP method. In this response, two trees were utilized, and various functions were considered to predict the output variable Gf using the input variables stated in the preceding sections.

**Fig. 8:**
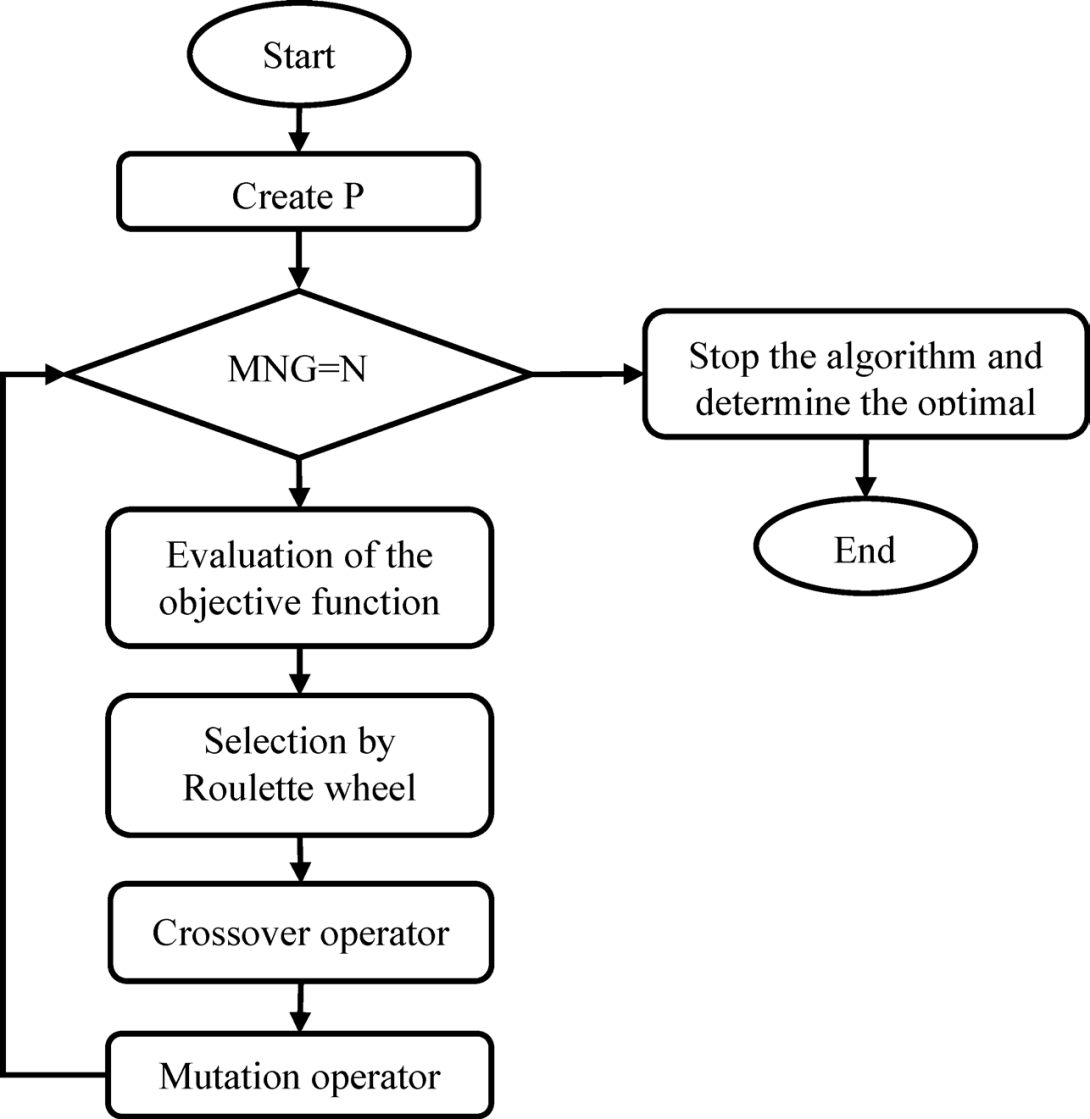
Flowchart of the MGGP technique.

Figure 8 illustrates the model development process using the MGGP method. The process begins by generating an initial population of responses with a size of P. When the number of iterations (N) is below the maximum number of generations (MNG) for the method, the population undergoes evaluation by the objective function. After evaluating the population using the objective function, a set of parents was selected based on the Roulette wheel cycle. These parents are then subjected to modifications using the crossover and mutation operators. The main aim of these modifications is to minimize the objective function, which represents the error rate between the model output and the data. These alterations are applied to the population in multiple repetitions, ensuring that the number of repetitions matches the predefined maximum value. By doing so, the optimal answer is aimed at being arrived at through this iterative process. To carry out this method, the MATLAB programming language is utilized, following the approach described in previous studies^47,48^.

To achieve prediction models with both high accuracy and low complexity, it is crucial to fine-tune the hyperparameters of the model. The key hyperparameters that significantly impact the complexity and accuracy of the model are population size, number of generations, maximum number of genes allowed in an individual, and maximum tree depth. The adjustment of these parameters has been carried out using a trial and error method, taking into consideration various modes based on past research, as shown in Table 7. By running the MATLAB software with the different modes presented in Table 7, the best-performing configuration was identified, resulting in the final model with optimal parameters. This process was repeated for all six datasets mentioned in the dataset section.

**Table 7:** Parameters setting for tuning the MGGP.

Variable ID	Settings
Population size	100, 200, 500
Number of generations	100, 200, 500
Maximum number of genes allowed in an individual	1–3
Maximum tree depth	1–3
Tournament size	12
Elitism	0.01
Crossover events	0.85
High-level crossover	0.2
Low-level crossover	0.8
Mutation events	0.1
Sub-tree mutation	0.9
Function set	Exp, Cube,$\sqrt {}$, $\times$, $+$, $-$,$/$

In summary, the approach involved carefully selecting hyperparameters using trial and error with insights from previous research^49,50^, ultimately leading to the creation of a highly accurate and efficient prediction model for each of the six datasets.

## Results and discussion

### Crack propagation in mode I

**Fig. 9:**
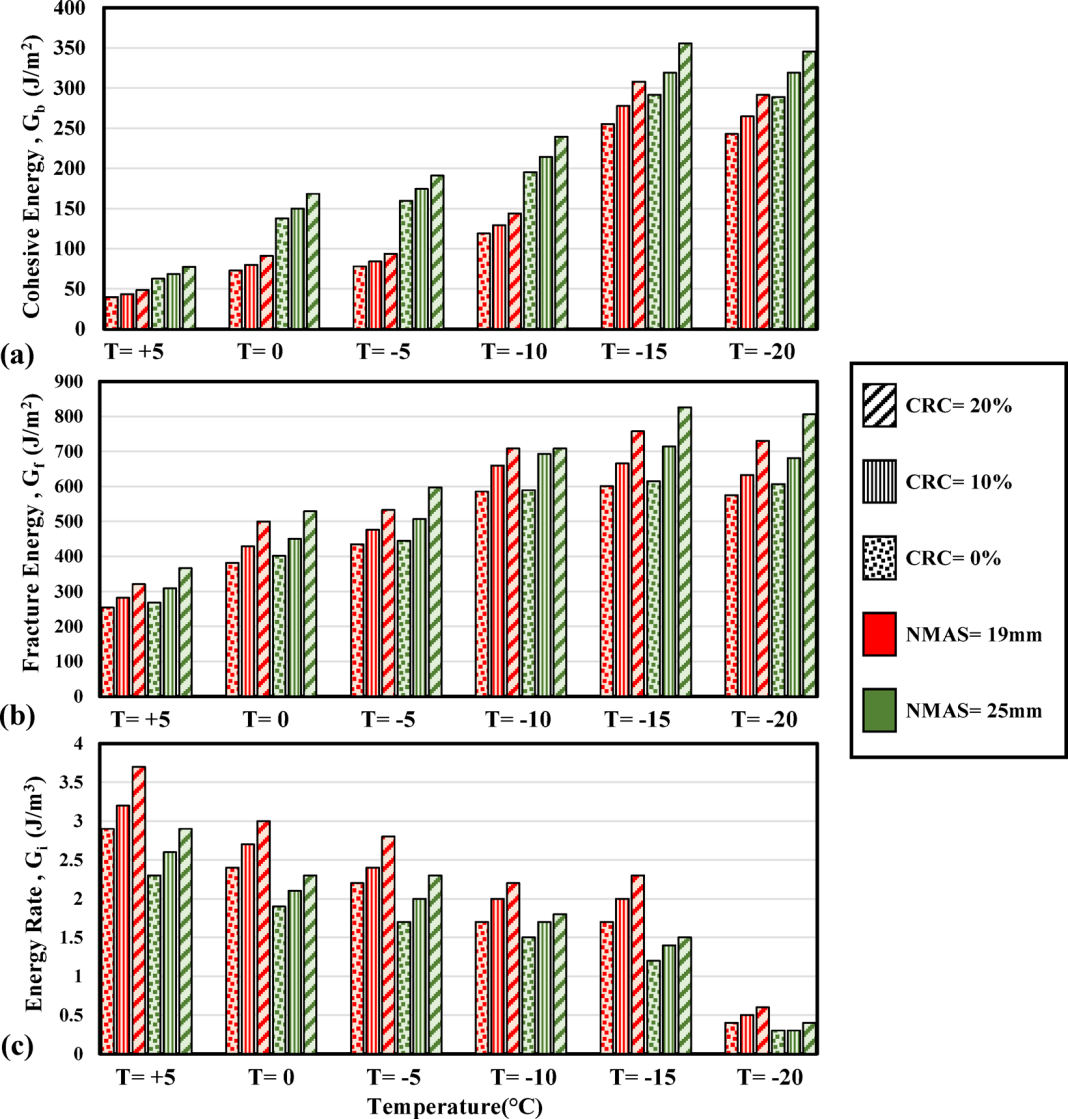
Variations of crack propagation parameters with temperature.

The three crack propagation parameters are derived against variations of temperature within the scope of this research and the plots are depicted in Fig. 9a–c. According to Fig. 9a, as the temperature is reduced the mixture develops higher toughening from + 5 °C to − 15 °C in this research and hence, the energy dissipated in blunting the crack tip is also increased. A higher initial toughness of the mixtures can be noted for 10% and 20% crumb rubber modified samples. As the temperature is reduced to below − 15 °C, lower energy will be required to blunt the crack tip as the mixture is undergoing a brittle fracture, and therefore, lower cohesive energy magnitudes are obtained for all the mixtures at − 20 °C than those in − 15 °C. The increase in the initial toughness of the mixtures is a cause of the stiffening of the binder as the temperature is reduced. The visible crack is initiated in the aggregate-binder interface where micro-voids are present. The interface is weak at lower temperatures and is strengthened as the binder is stiffened due to the drop in the temperature. Considering Fig. 9b, it can be observed that reduction of temperature has increased the fracture energy of the mixtures. However, at each temperature level, incorporating crumb rubber has increased the fracture energy by creating reinforcing particles inside the matrix and dissipating higher magnitudes of energy to attain the fracture of the mixture. Substantial brittleness of the mixture leads to lower fracture energy magnitudes for − 20 °C compared to − 15  °C. As described in Section “Crack propagation parameters and the R-curve method”, the energy rate parameter is principally addressing the post-peak fracture trend of the mixture dominated by the unstable crack propagation phase. In this regard, according to Fig. 9c, at higher temperatures in this research, higher energy rates can be observed while crumb rubber addition exhibits a positive effect as well. This can be attributed to more significant viscous dissipations after the peak load at higher temperatures as the binder is fairly nonelastic. As the temperature is reduced, the elasticity of the binder is increased and the viscous dissipations are declined which will lead to a lower rate of energy dissipation per unit extension of the crack in the mixture.

Based on Fig. 9a,b it can be observed that unmodified and crumb rubber modified mixtures with NMAS 25 mm are exhibiting higher magnitudes of fracture energy and also the energy required to form the initial crack surfaces. This agrees with the findings of Yang and Braham^37^. However, it can be noted from Fig. 9c that the post-peak resistance of the mixtures in terms of the energy dissipated to advance the growing crack declined as the nominal aggregate size increased. Lower aggregate size in the mixture will lead to further macro scale crack meandering in crossing aggregate-binder interface for the growing crack to cause a plastic wake and extend and hence higher energy will be dissipated while for larger NMAS values, larger aggregate could cause further rapid extension of a crack in the interface zone at low temperatures. Therefore, increasing the nominal maximum aggregate size in the mixtures is increasing the tendency for unstable crack growth leading to an abrupt failure of the mixture.

**Fig. 10:**
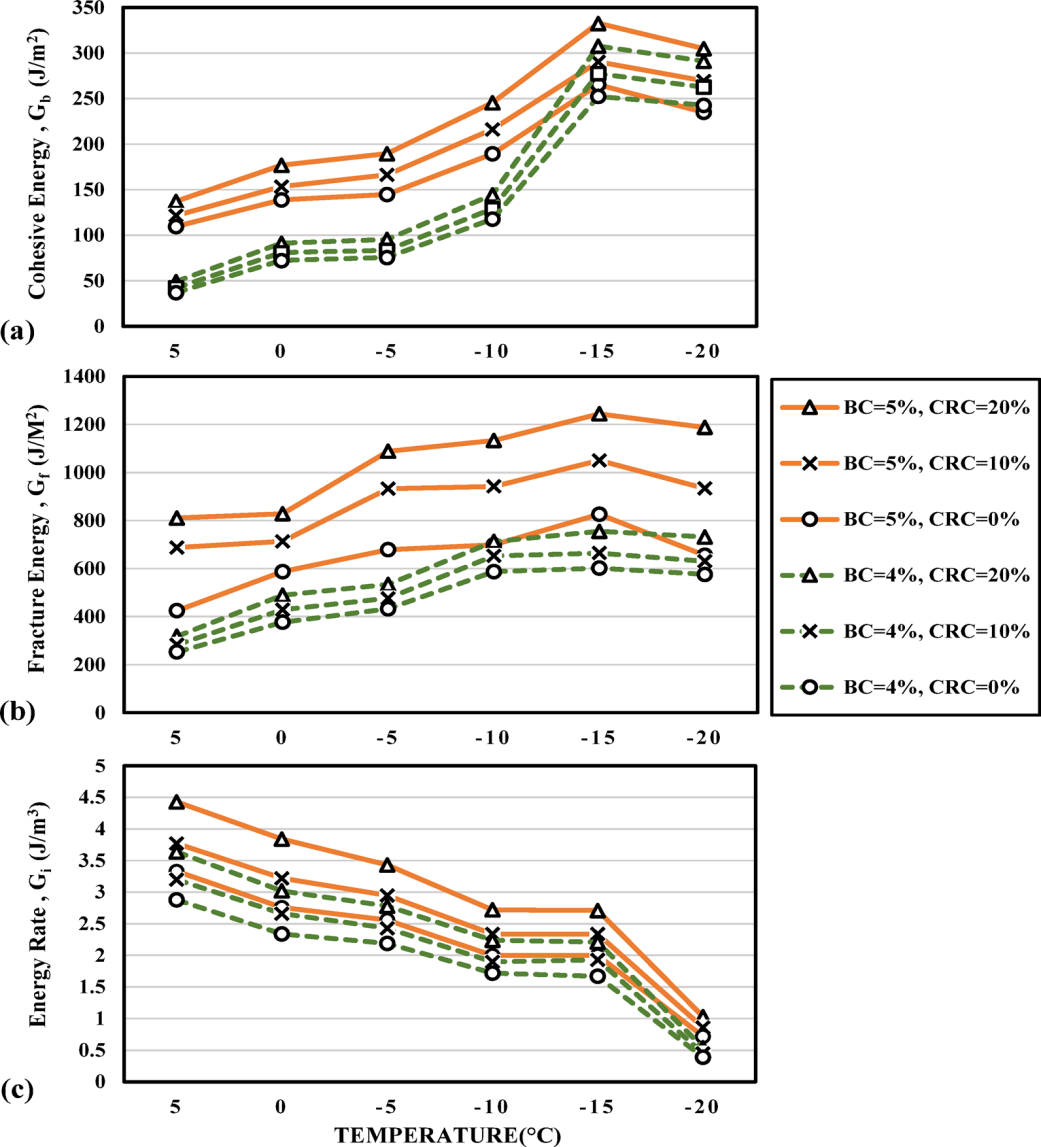
Variations of crack propagation parameters of the mixtures with binder contents.

The effect of binder content on the crack propagation energy parameters is depicted in Fig. 10a–c for mode I fracture. For better clarity, mixtures with 4.5% binder content are not presented in the charts and only those with the minimum and maximum binder content (4% and 5% respectively) are shown. The positive effect of increasing the binder content (0.5% from the optimal 4.5% obtained by the Marshall method) has improved resistance to crack initiation (higher G_b0_ values as in Fig. 10a) as well as increasing the total fracture energy of the mixture. This complies with the findings of Ahmad and Baigri^51^ who detected higher magnitudes of J-integral by increasing the binder content. Moreover, despite the brittleness of the mixture substantially increasing as the temperature is reduced, increasing the binder content is effective in maintaining a higher energy rate in the post-peak region. In other words, increasing the binder content above the optimal level has mitigated the unstable crack propagation tendency in the mixtures leading to greater post-peak resistance against crack propagation (Fig. 10c).

### Transition of crack propagation parameters from mode I to mixed-mode (I/II)

**Fig. 11:**
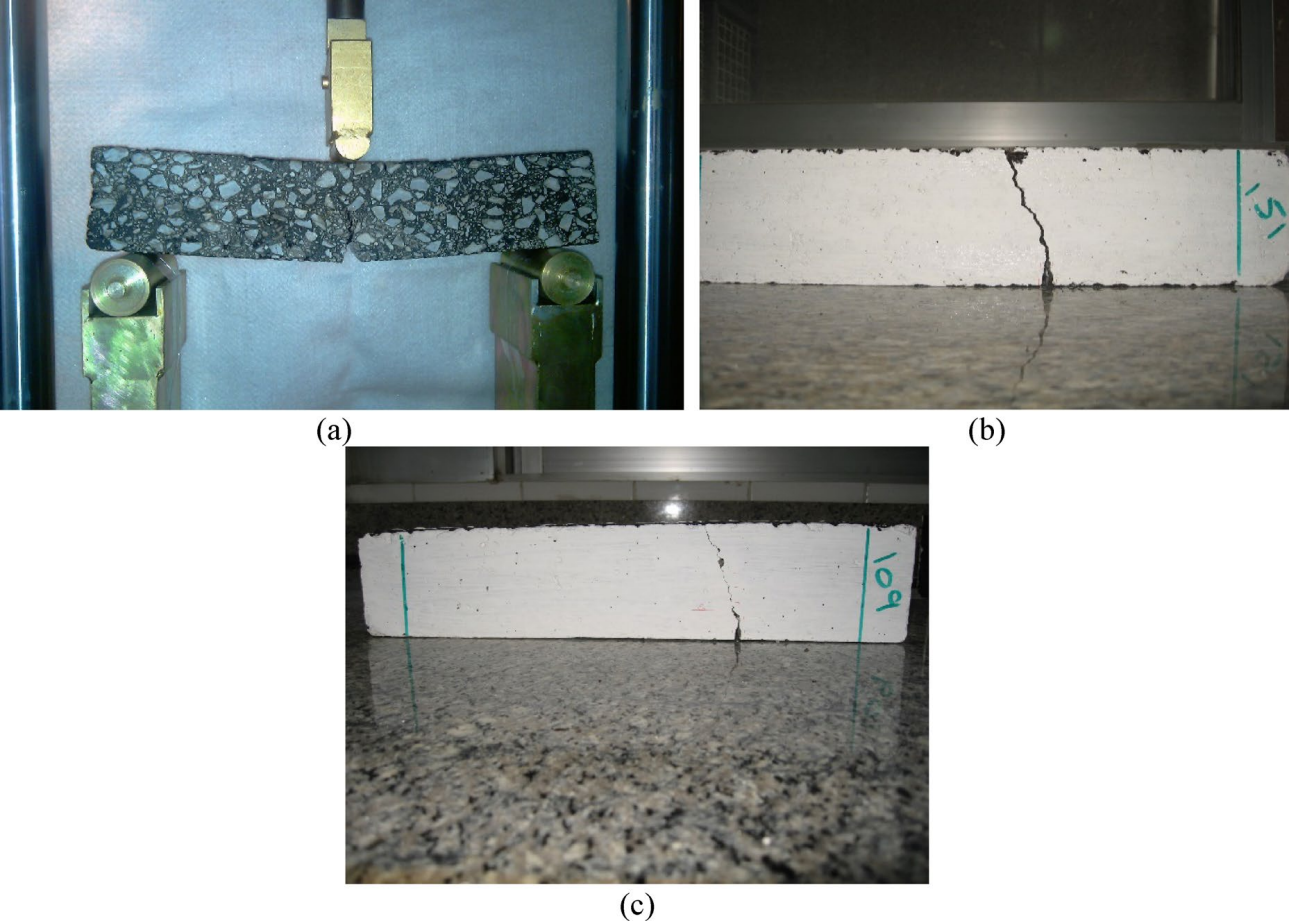
Sample comparison between (**a**) mode I fracture and (**b**) mixed-mode (I/II) with γ = 0.3, and (**c**) γ = 0.47 and crack path deviation towards the centerline.

As detailed in previous sections, the shearing component was induced in the AC beam specimens by fabricating the mechanical notch with normalized offset values of 0.3 and 0.47 from the centerline of the beam. For each SE(B) test in mixed-mode (I/II), visible cracking and fracture of the specimen were initiated from the notch tip. However, either instantly from the notch tip, or after vertical growth, all the cracks deviated towards the centerline of the beam (Fig. 11) regardless of temperature, and mixture properties. The growing cracks propagate predominantly in the interface zone between the aggregate and the binder^52,53^ up to a temperature level of − 10 °C while breakage of the aggregate can be seen as the temperature is dropped to below − 15 °C. Tables 8 and 9 present the test results for lime and siliceous aggregates, respectively, with parameters set to B = 4.0% and CRC = 10%.

**Table 8:** Crack propagation parameters in mode I and mixed-mode (I/II) for mixtures with lime aggregate.

NMAS (mm)	Offset (mm)	γ	Temperature (°C)	Cohesive energy (J/m^2^)	C.V	Fracture energy (J/m^2^)	C.V	Energy rate (J/m^3^)	C.V
19	0	0	5	80.204	0.36	537	0.21	3.95	0.35
			0	164.015		760		3.36	
			− 5	199.648		895		3.08	
			− 10	250.3		916		2.53	
			− 15	310.368		1099		2.42	
			− 20	272.783		990		0.94	
	48	0.3	5	206.279	0.30	1094	0.23	4.72	0.35
			0	246.885		1191		3.97	
			− 5	434.698		1767		3.66	
			− 10	467.032		1831		2.85	
			− 15	487.401		2168		2.88	
			− 20	471.042		1936		1.13	
	75	0.47	5	218.918	0.28	1237	0.24	9.96	0.37
			0	378.658		1490		8.35	
			− 5	386.205		1737		7.90	
			− 10	527.029		2012		5.85	
			− 15	562.136		2565		5.71	
			− 20	538.66		2214		2.26

**Table 9:** Crack propagation parameters in mode I and mixed-mode (I/II) for mixtures with siliceous aggregate.

NMAS (mm)	Offset (mm)	γ	Temperature (°C)	Cohesive energy (J/m^2^)	C.V	Fracture energy (J/m^2^)	C.V	Energy rate (J/m^3^)	C.V
19	0	0	5	73	0.34	475.23	0.20	2.21	0.37
			0	136		695.63		2.11	
			− 5	223		729.66		1.94	
			− 10	223		801.00		1.46	
			− 15	255		981.99		1.32	
			− 20	235		835.76		0.48	
	48	0.3	5	92	0.36	660.04	0.20	2.72	0.38
			0	185		986.34		2.63	
			− 5	296		1007.08		2.30	
			− 10	330		1068.57		1.78	
			− 15	345		1342.09		1.66	
			− 20	337		1123.64		0.57	
	75	0.47	5	126	0.35	1011.46	0.20	3.49	0.33
			0	258		1374.01		3.49	
			− 5	419		1520.32		2.98	
			− 10	434		1596.26		2.32	
			− 15	473		2011.42		2.20	
			− 20	432		1734.50		1.08	

**Fig. 12:**
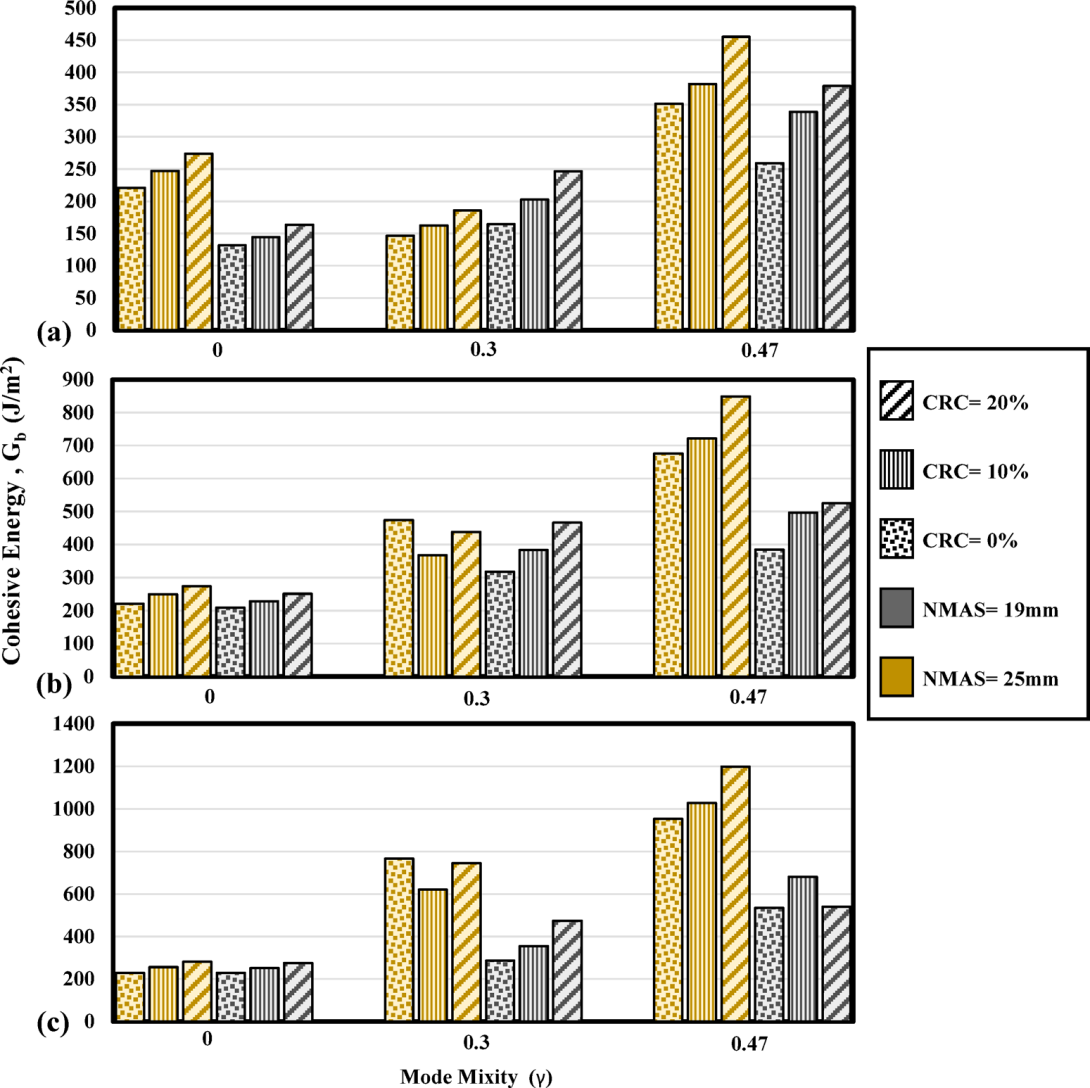
Effect of crumb rubber modification and mode mixity variation on cohesive energy of the mixtures at (**a**) 0 °C, (**b**) − 10 °C, and (**c**) − 20 °C.

Figure 12 shows the energy required to blunt the crack tip and initiate a visible crack for mode I and mixed-mode (I/II) conditions. As the mode mixity is increased from 0 to 0.3, the cohesive energy is increased. Samples with mode mixity values of 0.3 and 0.47 require higher energy dissipations to form cohesive surfaces around the notch tip. Meantime, as can be seen from Fig. 12a,b, reducing the temperature has a tangible effect in amplifying the resistance against crack initiation in mixed-mode (I/II). Strengthening of the bonding between the aggregate and the binder as the temperature is reduced plays an effective role in resistance against the formation of microvoids and cohesive surfaces in the notch tip.

**Fig. 13:**
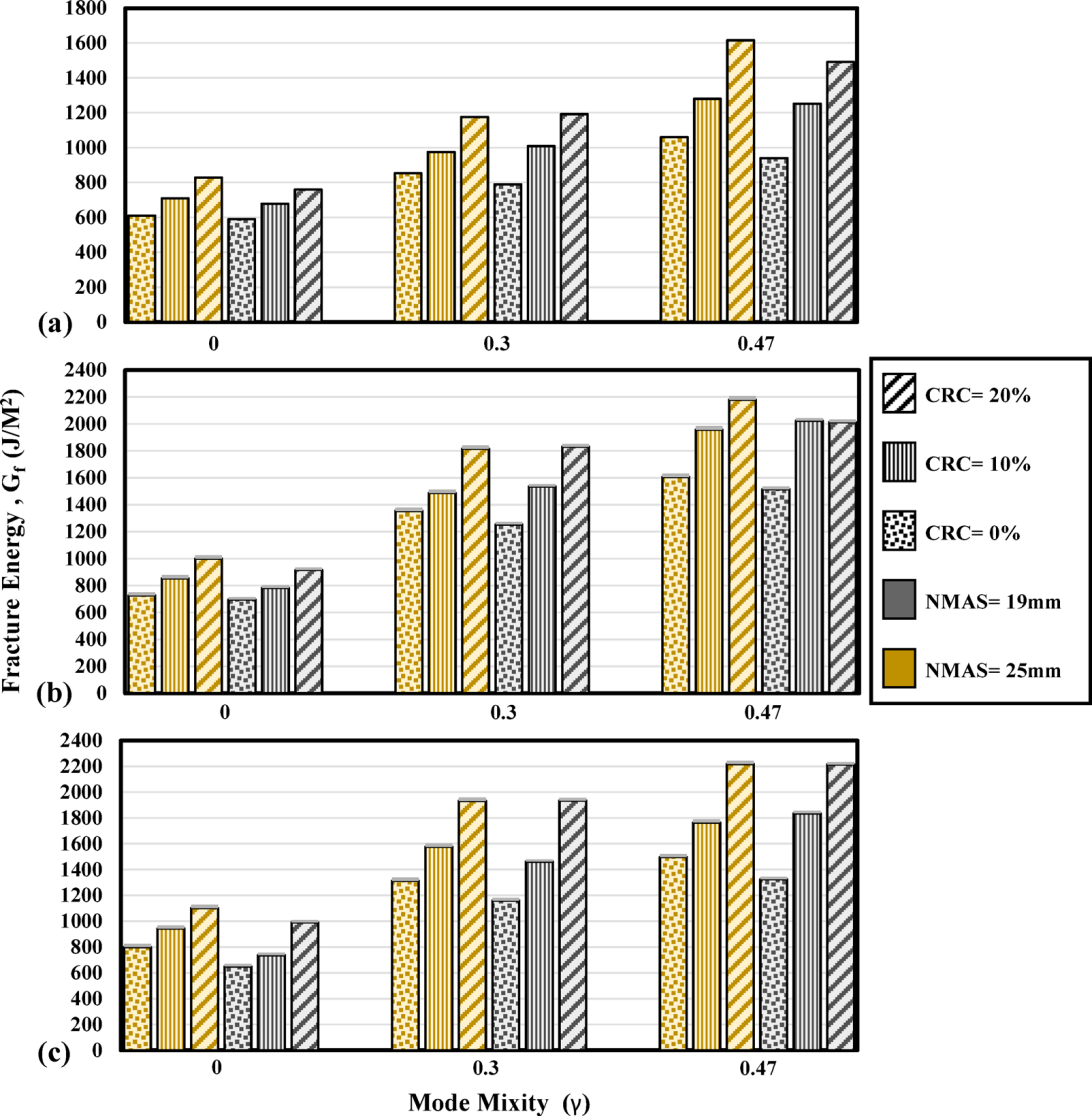
Effect of crumb rubber modification and mode mixity variation on fracture energy of the mixtures at (**a**) 0 °C, (**b**) − 10 °C, and (**c**) − 20 °C.

As can be seen from Fig. 13, increasing the notch offset from 0 to 0.3 has increased the total fracture energy of the mixture. Furthermore, as the offset value is increased, associated with higher induction of the shearing component, higher energy is required for the fracture of the specimen. For each mode mixitiy, it can be observed from Fig. 13 that as the temperature is reduced, higher stiffness of the binder and the bonds between the aggregate and the binder increase the fracture energy up to a temperature of − 20 °C where high brittleness dominates the fracture trend. Greater bonding can be obtained as the volume of bitumen is increased in the mixture, hence, mixtures with higher binder contents are exhibiting higher fracture energy magnitudes. In mode I condition, mixtures with NMAS 19 mm showed slightly lower fracture energy magnitudes than those with NMAS 25 mm and it can be seen from the literature that the effect of aggregate size on the fracture of the mixtures in mode I is subject to uncertainty and discrepancy as also emphasized by Yang and Braham^37,54^. Considering mixed-mode (I/II) fracture, it can be seen that the fracture energy magnitudes of the mixtures are substantially higher as the mode mixity is increased (Fig. 13) and the positive effect of using larger aggregate sizes can be confirmed for mixed-mode (I/II) fracture. Brittle fracture is dominant as the temperature reaches − 20 °C for all mode mixity values. As a result, the fracture energy magnitude for reference mixtures in γ = 0.47 at − 20 °C, is 1500 J/m^2^ and at − 15 °C is 1523 J/m^2^. In this state, mixtures with 10% crumb rubber modification are not exhibiting a tangible difference in the fracture energy. However, as the crumb rubber incorporation reaches 20%, the fracture energy magnitude for the mixtures at − 20 °C stands highest.

**Fig. 14:**
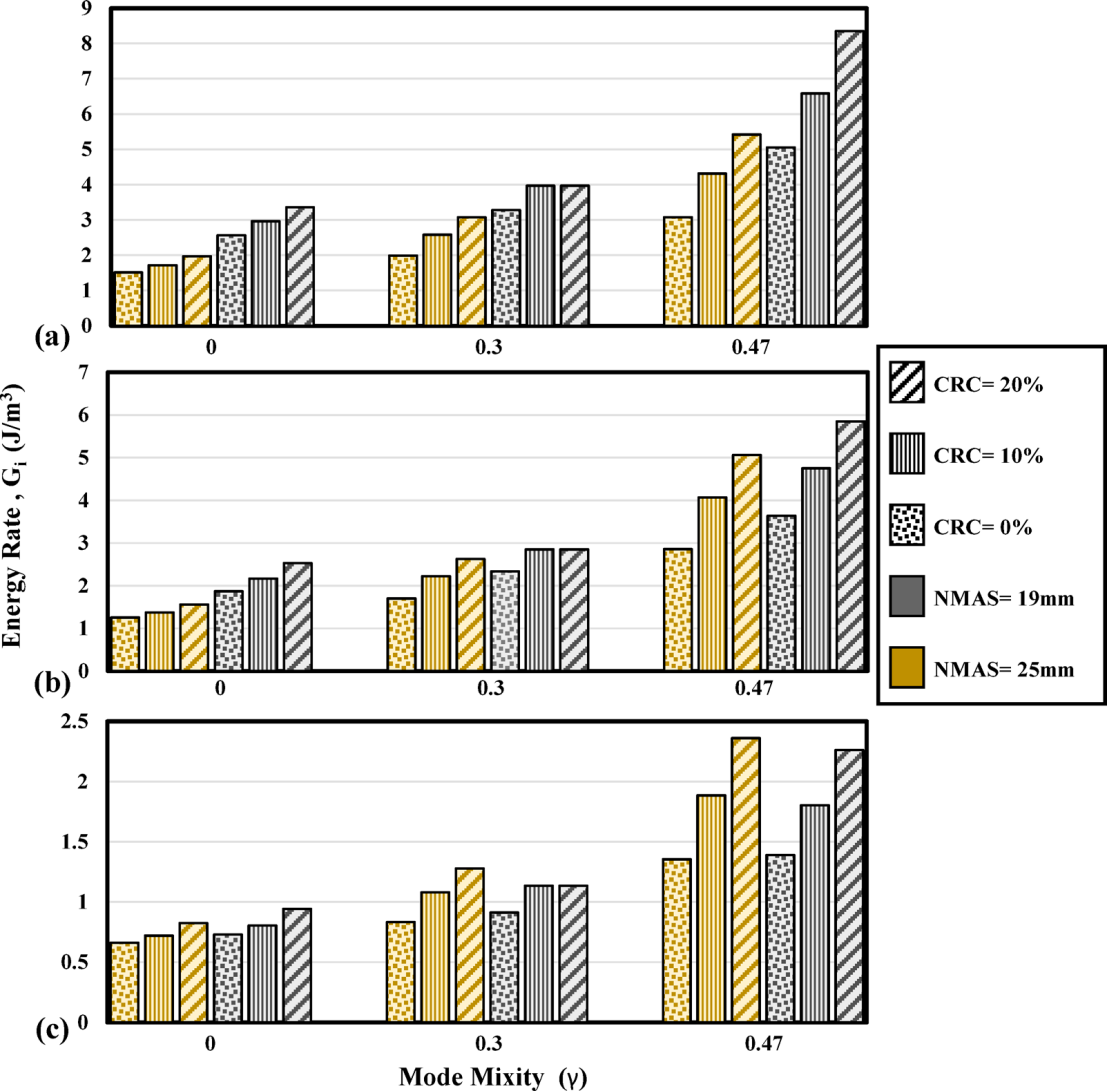
Effect of crumb rubber modification and mode mixity variation on the energy rate of the mixtures at (**a**) 0 °C, (**b**) − 10 °C, and (**c**) − 20 °C.

The post-peak fracture of the specimens with an induced shear mechanism is investigated using the energy rate parameter in this research. The energy dissipated per each increment of the crack growth is addressed using this parameter. Tables 8 and 9 are presenting crack propagation parameters for the mixture in different mode mixities. Considering the energy rate, for mode I condition, the energy rate is significantly reduced as the temperature is dropped. The energy rate is 0.94 J/m^3^ for the reference mixture with lime aggregate at − 20 °C and 0.48 J/m^3^ for the reference mixtures with siliceous aggregate. However, for the same mixtures, having a mode mixity of 0.3 the energy rate reaches values of 1.13 J/m^3^ and 1.66 J/m^3^ respectively, and 2.26 J/m^3^ and 1.08 J/m^3^ for γ = 0.47. This is indicating that as the sample is subject to mixed-mode (I/II) loading, i.e., the presence of tensile and shear mode fracture, higher resistance against crack propagation in the post-peak region can be expected from the mixtures (Fig. 14).

### Regression modeling

To create a regression model between the input variables and outputs, in the first step, the effect of each input variable on outputs was evaluated using analysis of variance (ANOVA) and pareto chart of standardized effects in Minitab software. In the Pareto analysis, the magnitude of the effect of inputs on observation was determined as follows^55,56^:

6 $$P.E_{i} = \frac{{c_{i}^{2} }}{{\sum\limits_{{}}^{{}} {c_{i}^{2} } }} \times 100\quad i \ne 0$$

where: P.${\text{E}}_{\text{i}}$: Percentage effect of the i_th_ term of the model, ${\text{C}}_{\text{i}}$: The coefficient of the i_th_ term of the model.

**Fig. 15:**
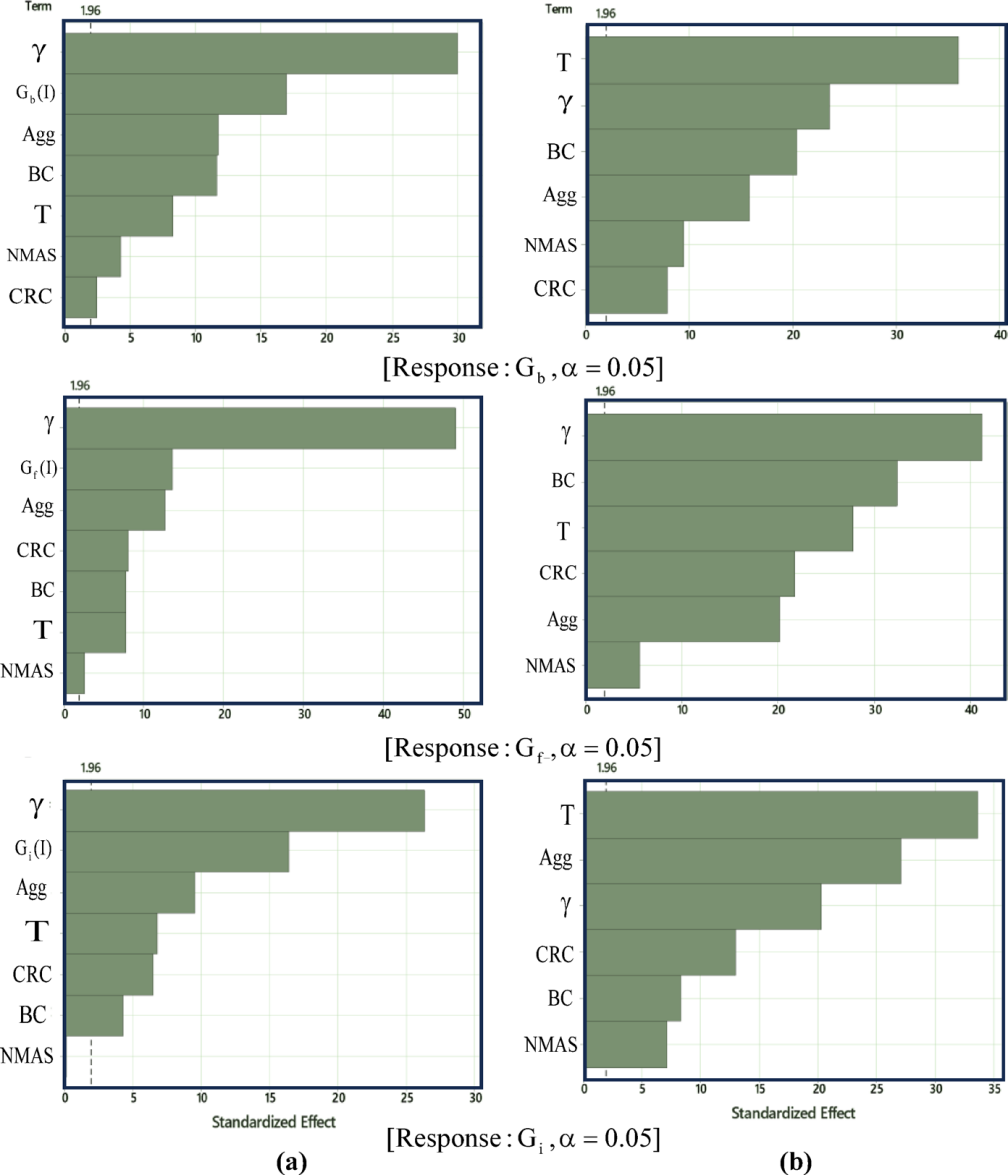
Pareto analysis on (**a**) dataset 1, and (**b**) dataset 2.

According to the Pareto analysis of effects, as illustrated in Fig. 15, it is evident that, as anticipated, the value of the parameter '$\gamma$' and the outcome associated with Mode I exert the most substantial influence on the mixed-mode (I/II) response. This suggests that the interplay between these factors is critical in determining the behavior of the system under mixed-mode loading conditions. On the other hand, the analysis reveals that NMAS does not significantly impact the G_i_, indicating that this particular parameter can be considered negligible in this context. Conversely, all other input parameters demonstrate a significant influence on the output variables.

As detailed in Section “Dataset”, the Box-Cox transformation was employed to address the non-normal distribution of the output data, effectively converting it into a more normal shape. This transformation is crucial for enhancing the efficiency and reliability of the prediction model by mitigating issues like non-normality of errors and heteroscedasticity, which could otherwise compromise the model’s performance. The application of this technique resulted in the determination of optimal power transformation parameters, with values of 0.113149 J/m^2^ for G_b_, 0.16973 J/m^2^ for G_f_, and 0.268088 J/m^3^ for G_i_.

To model the relationship between the experimental responses and variations in the considered factors, several model structures were evaluated, including linear, quadratic (second-order), etc. ANOVA was employed to assess the significance of each term within the model structure for modeling the experimental data. The results of the ANOVA on linear model structure is presented in Table 10.

**Table 10:** ANOVA results on datasets.

Variable	G_b_				G_f_				G_i_			
	Dataset 1		Dataset 2		Dataset 1		Dataset 2		Dataset 1		Dataset 2	
	Coef	*P* Value	Coef	*P* Value	Coef	*P* Value	Coef	*P* Value	Coef	*P* Value	Coef	*P* Value
Agg	− 0.05029	< 0.05	− 0.0786	< 0.05	− 0.10364	< 0.05	− 0.16294	< 0.05	− 0.0968	< 0.05	− 0.22205	< 0.05
NMAS	0.01857	< 0.05	0.04708	< 0.05	0.01839	< 0.05	0.04575	< 0.05	0.00129	< 0.05	− 0.05897	< 0.05
B_r	0.06832	< 0.05	0.12384	< 0.05	0.1288	0.011	0.32035	< 0.05	0.3752	0.865	0.0844	< 0.05
CR-r	0.01313	0.013	0.04817	< 0.05	0.0976	< 0.05	0.21478	< 0.05	0.0598	< 0.05	0.1305	< 0.05
T-r	− 0.0946	< 0.05	− 0.26158	< 0.05	− 0.1354	< 0.05	− 0.3286	< 0.05	0.1322	< 0.05	0.4051	< 0.05
γ_r	0.14426	< 0.05	0.1413	< 0.05	0.41193	< 0.05	0.40229	< 0.05	0.2124	< 0.05	0.20178	< 0.05
G_0_	0.3484	< 0.05	–	–	0.6273	< 0.05	–	–	0.6783	< 0.05	–	–

Accoding to Table 10, all variables in the two datasets have a significant effect on G_f_ and G_b_. NMAS as an insignificant terms (*p* value > 0.05) in dataset 1 of G_i_ was removed, resulting in a simplified polynomial model for each response. The equations of the final modelsare presented in Eqs. (7) through (12) Eqs. 3–5.

dataset 1-1:

7 $$G_{f}^{0.16973} = \begin{array}{*{20}l} \begin{gathered} 2.7296 - 0.10364 AT + 0.01839 NMAS \, + 0.1288 B + 0.0976 CRC - 0.1354 T \hfill \\ + 0.41193 \gamma + 0.6273 G_{f0} \hfill \\ \end{gathered} \hfill \\ \end{array}$$

dataset 2-1:

8 $$G_{f}^{0.16973} = \begin{array}{*{20}l} \begin{gathered} 2.9509 - 0.1629AT + 0.0458 NMAS \, + 0.3204 B + 0.2148\,CRC - 0.3286 T \hfill \\ + 0.4023 \gamma \hfill \\ \end{gathered} \hfill \\ \end{array}$$

dataset 1-2:

9 $$\begin{aligned} G_{b}^{{0.113149}} & = 1.6786 - 0.05029AT + 0.01857NMAS + 0.06832B + 0.01313CRC \\ & \quad - 0.0946T + 0.14426\gamma + 0.3484G_{{b0}} \\ \end{aligned}$$

dataset 2-2:

10 $$\begin{aligned} G_{b}^{{0.113149}} & = 1.8486 - 0.0786AT + 0.0471NMAS + 0.1238B + 0.0482CRC \\ & \quad - 0.2616T + 0.1413\gamma \\ \end{aligned}$$

dataset 1-3:

11 $$\begin{aligned} G_{i}^{{0.268088}} & = 0.8016 - 0.09729AT + 0.03770B + 0.06005CRC + 0.1334T \\ & \quad + 0.21201\gamma + 0.6739G_{{i0}} \\ \end{aligned}$$

dataset 2-3:

12 $$\begin{aligned} G_{i}^{{0.268088}} & = 0.9295 - 0.2221AT - 0.0589{\mkern 1mu} NMAS + 0.0844B + 0.1305CRC \\ & \quad + 0.4051T + 0.2018\gamma \\ \end{aligned}$$

### MGGP modeling

**Fig. 16:**
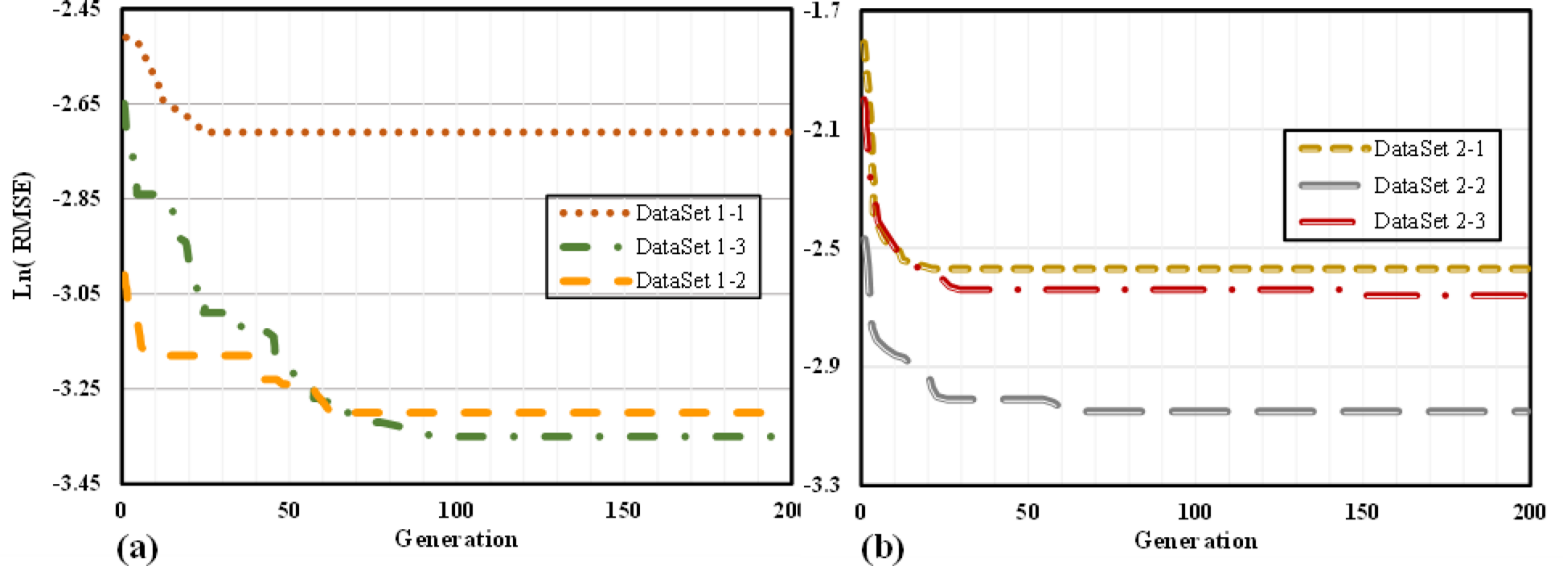
Model training process to predict (**a**) dataset 1, and (**b**) dataset 2.

In this section, the intricate mathematical relationships established by the MGGP method between the input and output variables are thoroughly explored. Initially, an overview of the training process applied to various datasets is provided, with the process visually illustrated in Fig. 16. In Fig. 16a, the results of the first dataset, which includes mode I of fracture as an input variable, are depicted. Conversely, Fig. 16b displays the results for the second dataset, where mode I fracture is excluded from the input variables. The x-axis represents the number of iterations during which the MGGP method was run to optimize the model, while the y-axis depicts the corresponding error values.

The error is observed to decrease steadily with increasing iterations during the early phase of training, signifying that the model is progressively learning and following the designated training path. After approximately 100 iterations, the error plateaus, indicating that the model has reached convergence and the training phase is considered complete. Notably, the larger range of G_f_ data accounts for the higher ln(RMSE) values in the G_f_ prediction datasets. However, this does not imply that the models inherently exhibit more error.

The final models generated represent an optimal balance between complexity and accuracy for each dataset. The equations corresponding to these optimal models are presented below, capturing the mathematical formulations developed for both datasets.

dataset 1-1

13 $$G_{{_{f} }}^{0.16973} = 0.053\left( {\delta + \sqrt {G_{f0}^{{}} } } \right)\left( {CRC + \gamma + 20.46} \right) - 0.04\left( {AT + 9.289} \right)\left( {2\gamma + 0.775} \right) + 2.605$$

dataset 2-1

14 $$G_{{_{f} }}^{0.16973} = 0.188\left( {B + CRC - AT - \exp \left( T \right)} \right) + 0.377\gamma - 0.132\left( {\exp \left( {T - B} \right) + \sqrt[4]{T}} \right) + 3.302$$

dataset 1-2

15 $$G_{{_{b} }}^{0.113149} = 0.068\left( {\gamma + \exp \left( \gamma \right) - AT \times \gamma } \right) + \frac{{8.845 \times 10^{15} \left( {B - T} \right)}}{{1.801 \times 10^{16} AT + 2.099 \times 10^{17} }} + 0.491\sqrt {G_{{_{b0} }}^{{}} } + 1.437$$

dataset 2-2

16 $$\begin{aligned} G_{{_{b} }}^{0.113149} & = 0.054\left( {NMAS - AT + CRC + \gamma + \exp \left( \gamma \right)} \right) - 0.004\left( {2T - B + 2.837} \right)^{3} \\ & \quad - 0.054 \times AT \times NMAS + 1.881 \\ \end{aligned}$$

dataset 1-3

17 $$G_{i}^{0.268088} = 0.9185\sqrt {G_{{_{i0} }}^{{}} } + 0.033 \times \gamma^{2} \left( {T + 2.588} \right)\left( {CRC - \exp \left( {AT} \right) + 3.545} \right) + 0.602$$

dataset 2-3

18 $$G_{i}^{0.268088} = 0.079\left( {CRC - NMAS + \gamma \times CRC + B^{2} } \right) + 0.226\left( {\sqrt T - AT + \sqrt[4]{T}} \right) + 0.158\gamma + 0.879$$

The variables in the above equations are defined in Section “Dataset”. Feature selection is a critical task in machine learning that involves selecting a subset of input variables that can accurately predict the output variable. The MGGP method performs feature selection using a trial-and-error approach, and selects a combination of variables that best predict the output variable. The variables selected by the MGGP method are shown in Table 11.

**Table 11:** Feature selection results for the developed models.

Output variable	Dataset ID	G_f0_ (Pa)	G_b0_ (Pa)	G_i0_ (Pa)	γ (%)	B (%)	CRC (%)	T (°C)	AT (°C)	NMAS (Hz)
G_f_ (Pa)	1–1	✓	×	×	✓	×	✓	×	✓	×
	2–1	×	×	×	✓	✓	✓	✓	✓	×
G_b_ (Pa)	1–2	×	✓	×	✓	✓	×	✓	✓	×
	2–2	×	×	×	✓	✓	✓	✓	✓	✓
G_i_ (Pa)	1–3	×	×	✓	✓	×	✓	×	✓	×
	2–3	×	×	×	✓	✓	✓	✓	✓	✓

**Fig. 17:**
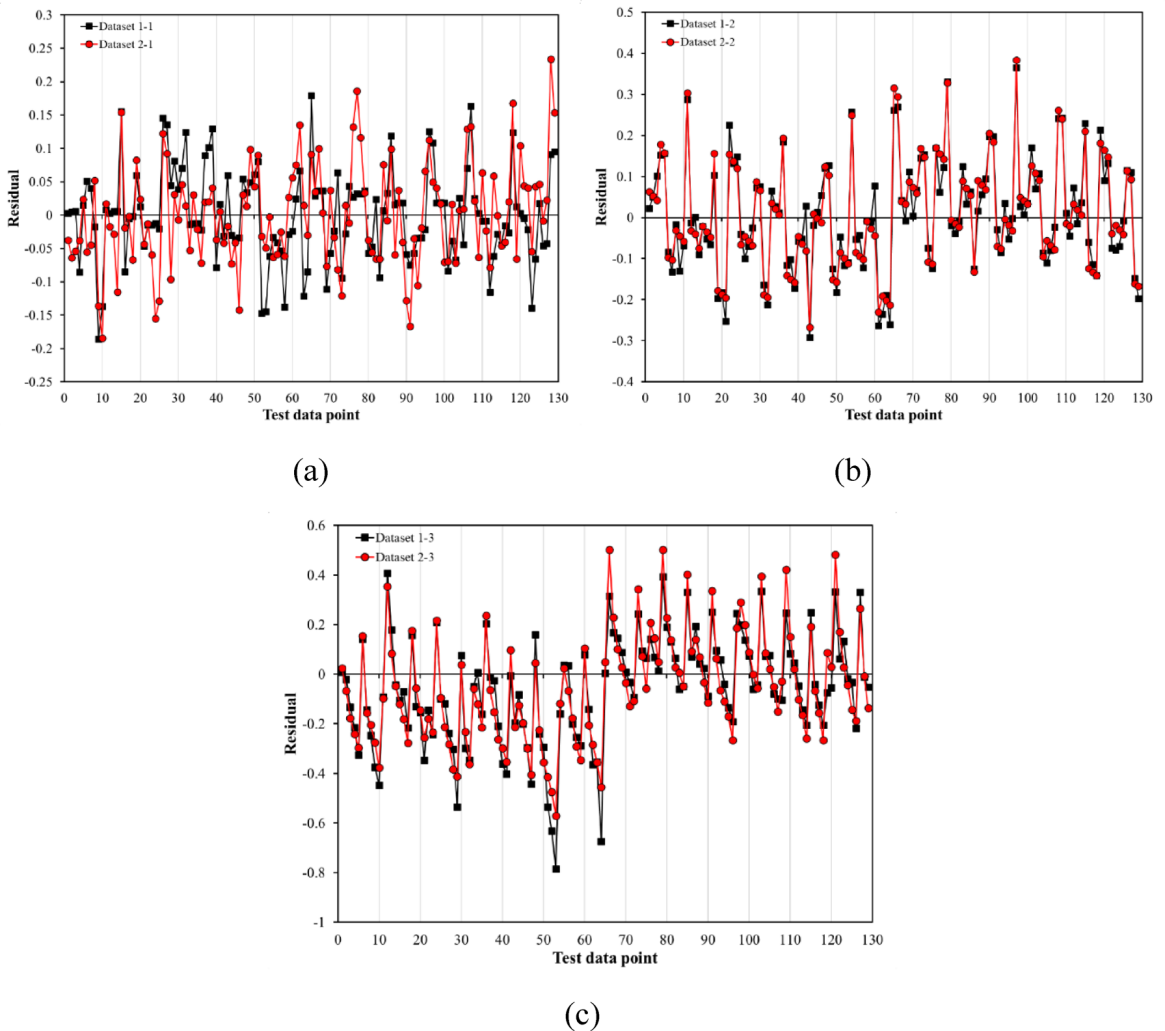
Evaluation of residuals for models (**a**) G_f_, (**b**) G_b_ and (**c**) G_i_.

The residuals of the test data were used to evaluate the validity of the developed models. Residuals are the difference between the actual and predicted values. The closer the residuals are to zero, the more accurate the model is. Figure 17 shows that the residuals of all models are close to zero, indicating that the models are accurate. The X-axis of Fig. 17 represents the test data points, and the Y-axis represents the difference between the test data and the predicted values, also known as the residuals. Additionally, the residuals are randomly distributed, with no particular pattern. Finally, datasets 1-1, 2-1, and 3-1, which use mode 1 as the input variable, have lower residuals than datasets 1-2, 2-2, and 3-2.

### Comparative analysis of regression and MGGP

The accuracy of the models has been evaluated using a variety of metrics, all of which are detailed in Eqs. (19–21) ^57,58^:

19 $$MAE = \frac{1}{n}\sum\limits_{i = 1}^{n} {\left( {\left| {T_{i} - \overline{T}_{i} } \right|} \right)}$$

20 $$MSE = \frac{{\sum\limits_{i = 1}^{n} {(T_{i} - P_{i} )^{2} } }}{n}$$

21 $$R^{2} = \left( {\frac{{\sum\limits_{i = 1}^{n} {\left( {T_{i} - \overline{T}} \right)\left( {P_{i} - \overline{P}} \right)} }}{{\sqrt {\sum\limits_{i = 1}^{n} {\left( {T_{i} - \overline{T}} \right)^{2} } \sum\limits_{i = 1}^{n} {\left( {P_{i} - \overline{P}} \right)} } }}} \right)^{2}$$

In these equations, "T" represents the actual target values, while "P" signifies the predicted values. "T̅" refers to the average value derived from the target data, and "P̅" denotes the mean of the predicted values generated by the model. Lastly, "n" stands for the total number of data points being considered in the analysis.

**Fig. 18:**
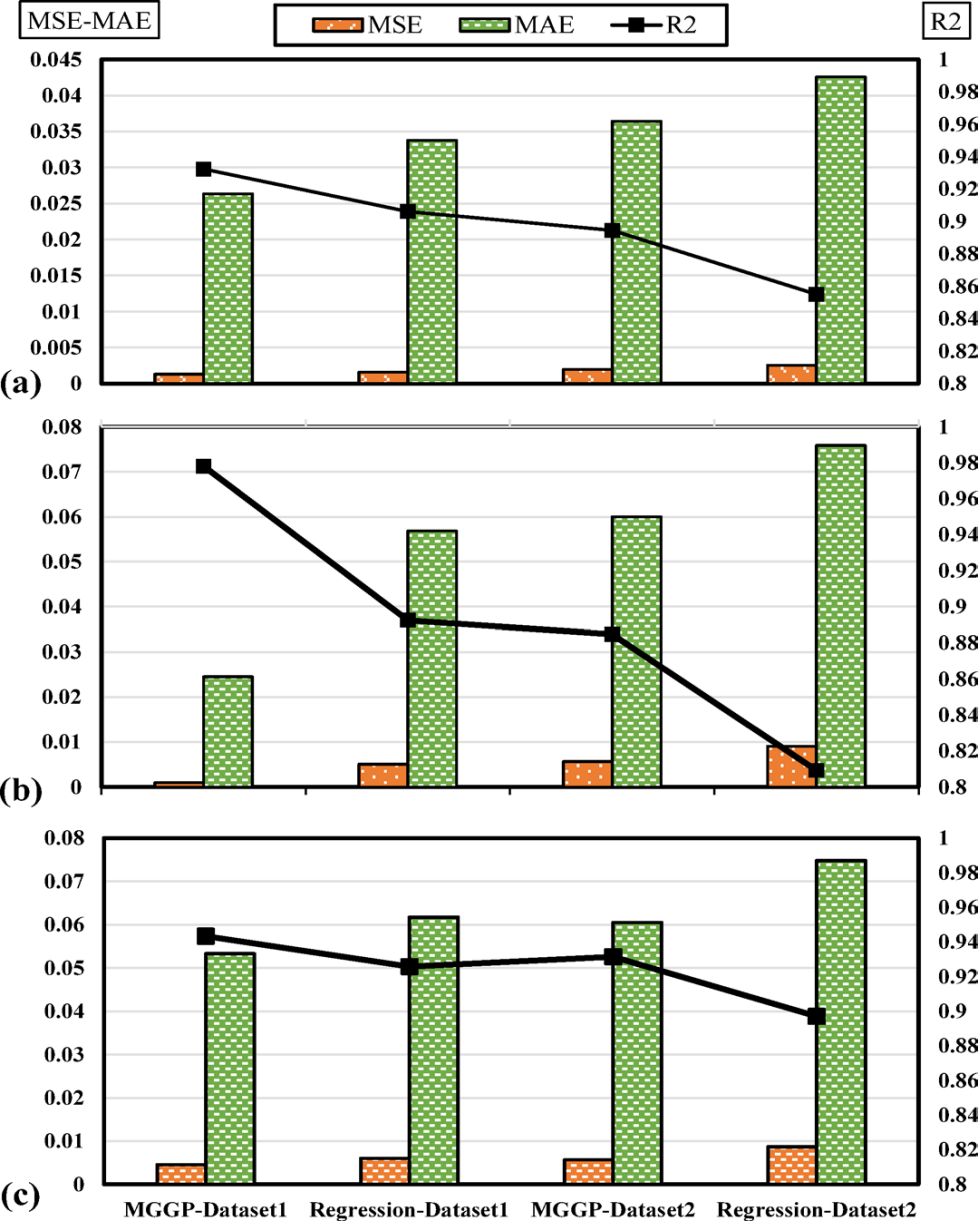
Evaluation of the accuracy of models (**a**) G_b_, (**b**) G_f,_ and (**c**) G_i_.

The reliability of the models has been evaluated using Fig. 18, which was created with test data to compare the performance of MGGP and regression techniques across the two datasets. The performance of the models is measured by two key factors: the magnitude of the R^2^ value and the reduction in MAE and MSE. Superior performance is indicated by higher R^2^ values and lower MAE and MSE scores.

Upon closer examination of Fig. 18, it is observed that all models exhibit very low error rates and achieve a high level of accuracy in predicting the output variables. These models are applicable in various scenarios. A notable advantage over methods, such as neural networks and random forests, is the ability to provide engineers with a practical formula that links the input and output variables, simplifying their application.

Figure 18 demonstrates that models developed using Dataset 1 and the MGGP approach consistently outperform their counterparts. On a broader scale, it is evident that Dataset 1 is more effective in predicting output variables than Dataset 2. This difference can be attributed to the inclusion of three energy parameters related to mode I as input variables, which play a crucial role in the enhanced performance of Dataset 1. Despite this, the results from both datasets are remarkably similar, even though Dataset 2 lacks these energy parameters. This indicates that Dataset 2 can serve as a cost-effective alternative to Dataset 1, which is more resource-intensive to collect. It is also worth noting that the MGGP method consistently delivers higher accuracy compared to the regression approach. This performance gap is largely due to MGGP’s flexibility in utilizing various functions and forms for output prediction, which sets it apart from traditional linear regression methods.

**Fig. 19:**
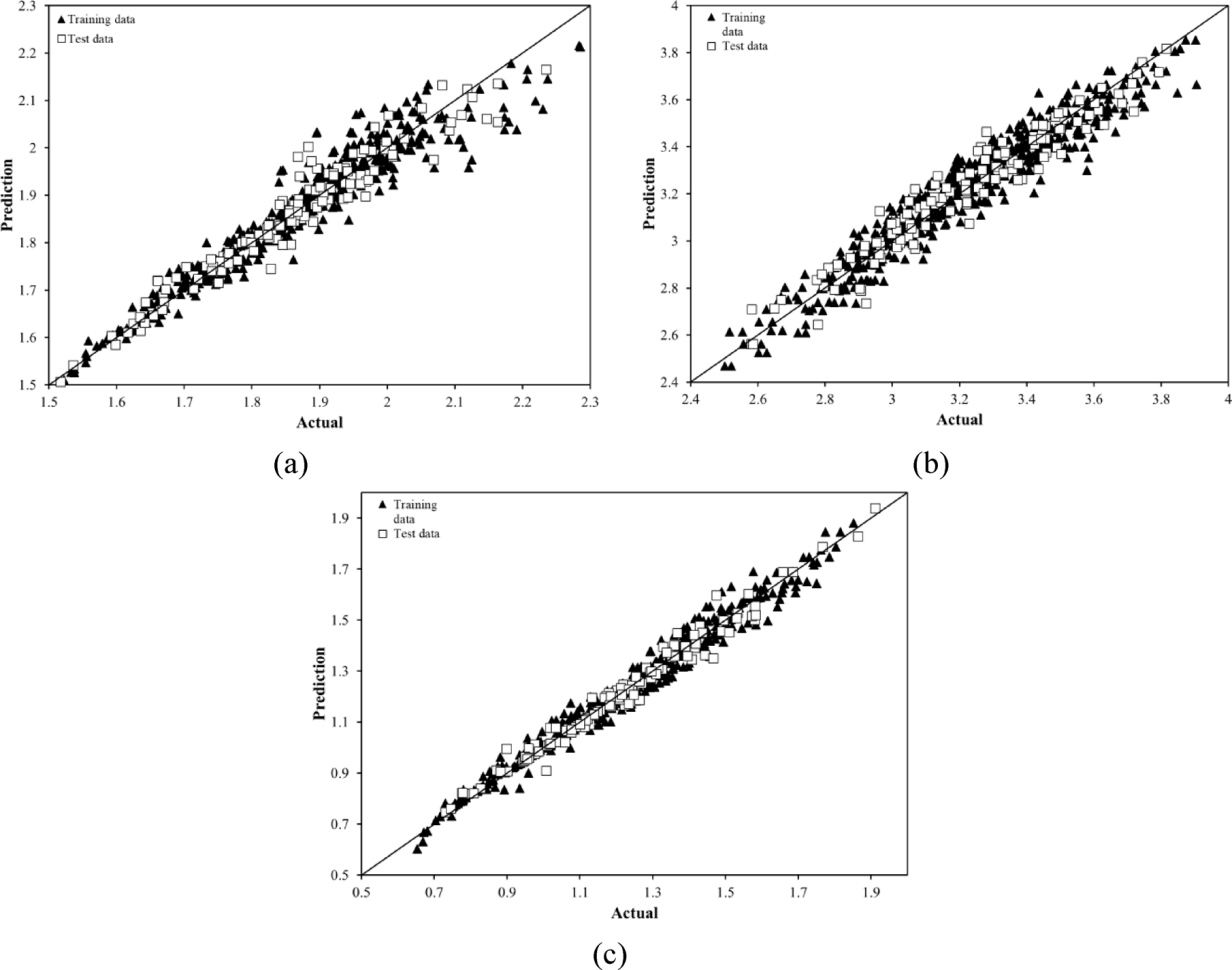
Visualization of the performance of MGGP models on dataset 1 for (**a**) G_b_, (**b**) G_f_ and (**c**) G_i_.

As mentioned earlier, the MGGP method showed significantly better performance than other methods when used with Dataset 1. Figure 19 displays a scatter plot highlighting the relationship between the actual data and the predictions, demonstrating the strong accuracy of the MGGP method on this dataset. The accuracy of the model’s predictions is evident in how closely the data points follow the Y = X reference line. Importantly, among the models developed, the G_f_ model has the largest error. This is clear from the way its data points stray further from the Y = X line compared to the other two models, underlining the stronger performance of the others.

### Comparison with conventional machine learning methods

To further evaluate the performance of the proposed MGGP model, a comparative analysis was conducted using several conventional machine learning methods. The models were trained and tested on Dataset 1, where Mode I crack propagation parameters were considered as input features. Since MGGP consistently outperformed linear regression across all target variables (G_b_, G_f_, and G_i_), only MGGP was selected for comparison with other machine learning models, namely Support Vector Regression (SVR), Random Forest (RF), and Artificial Neural Networks (ANN). The hyperparameters of all machine learning models were optimized using grid search to ensure fair and reliable performance. Table 12 shows the performance comparison between the proposed MGGP model and conventional machine learning algorithms, including SVR, Random Forest, and ANN, for predicting Mode I crack propagation parameters (G_b_, G_f_, and G_i_) using Dataset 1.

**Table 12:** Performance comparison of MGGP and conventional machine learning models.

Parameter	G_b_			G_f_			G_i_		
Model	R^2^	MSE	MAE	R^2^	MSE	MAE	R^2^	MSE	MAE
MGGP	0.9326	0.0013	0.0263	0.9433	0.0048	0.0533	0.9756	0.0012	0.0245
SVR	0.9949	0.0003	0.0115	0.9876	0.0010	0.0218	0.9441	0.0028	0.0375
RF	0.9834	0.0008	0.0196	0.9699	0.0025	0.0344	0.9823	0.0009	0.0228
ANN	0.9722	0.0014	0.0302	0.9173	0.0070	0.0658	0.9569	0.0022	0.0368

Overall, the results on the test datasets demonstrate strong predictive performance across all models. Although MGGP shows slightly lower accuracy compared to SVR and Random Forest in some cases, its major advantage is the ability to provide explicit mathematical expressions for predicting crack propagation parameters. This makes MGGP a more interpretable and practical approach, particularly in engineering contexts where transparency and insight into the model’s structure are important.

## Conclusions

The objective of this research was to develop an intelligent and effective model for predicting fracture parameters under mixed-mode (I/II) loading conditions in asphalt mixtures. In this study, asphalt mixtures containing varying percentages of crumb rubber, different temperatures, and other controlled laboratory conditions were subjected to SE(B) testing. A comprehensive dataset was subsequently generated, encompassing mode I crack propagation parameters (G_b0_, G_f0_, and G_i0_), crumb rubber content, aggregate type, binder content, nominal maximum aggregate size, temperature, and normalized offset ratio. Given the high cost of obtaining mode I fracture parameters, an independent dataset was created, excluding mode I fracture parameters, based on predictions of mixed-mode (I/II) fracture behavior. Machine learning techniques, including Multi-Gene Genetic Programming (MGGP) and regression, were then applied to both datasets to predict the mixed-mode (I/II) fracture parameters, yielding the following results:

- Increasing the notch offset from 0 to 0.3 enhances the total fracture energy of the mixture, indicating that higher induction of the shearing component requires more energy for fracture. Additionally, lower temperatures increase the fracture energy up to − 20 °C due to higher binder stiffness and bond strength, but significant brittleness dominates at this temperature. Mixtures with higher binder content and larger aggregate sizes show greater fracture energy, particularly under mixed-mode (I/II) fracture conditions. Brittle fracture is prevalent at − 20 °C, and while 10% crumb rubber modification shows minimal effect, 20% crumb rubber significantly increases fracture energy at this temperature.
- The study reveals that the energy rate parameter, which measures the energy dissipated per increment of crack growth, significantly increases under mixed-mode (I/II) loading compared to pure mode I. Specifically, the energy rate for lime aggregate mixtures at − 20 °C increases from 0.94 J/m^3^ in mode I to 2.26 J/m^3^ under mixed-mode with γ = 0.47, indicating higher resistance against crack propagation in the post-peak region when both tensile and shear modes are present.
- The results of the Pareto analysis for the regression model development indicate that the normalized offset ratio and mode I parameters exert the most significant influence on predicting mixed-mode (I/II) behavior.
- Both methods, regression and MGGP, developed for predicting fracture parameters in mixed-mode (I/II) show strong performance. However, the results demonstrate that the MGGP method offers superior accuracy compared to the regression approach across the two datasets. In the numerical test data, the R-squared values for the MGGP method range from 0.88 to 0.97, while the regression method yields R-squared values between 0.80 and 0.92.
- In the MGGP method, the difference in R^2^ values when mode I is included as an input versus when excluded is 0.04, 0.01, and 0.04 for G_b_, G_f_, and G_i_, respectively. This difference emphasizes the importance of precision in engineering problems and demonstrates that incorporating mode I leads to more accurate predictions within the MGGP framework.

## Data Availability

The datasets generated during and/or analysed during the current study are available from the corresponding author on reasonable request.
